# Life History and Fishing Aspects of the Deep-Sea Silver Scabbardfish *Lepidopus caudatus* in the Azores

**DOI:** 10.3390/biology11111619

**Published:** 2022-11-06

**Authors:** Gloria Mariño-Briceño, Wendell Medeiros-Leal, Ualerson Iran Peixoto, Mário Pinho, Régis Santos

**Affiliations:** 1 Okeanos–UAc Instituto de Investigação em Ciências do Mar, Universidade dos Açores, Rua Prof. Dr. Frederico Machado, 4, 9900-138 Horta, Portugal; 2 IMAR Instituto do Mar, Departamento de Oceanografia e Pescas, Universidade dos Açores, Rua Prof. Dr. Frederico Machado, 4, 9901-862 Horta, Portugal

**Keywords:** population structure, growth parameters, mortality, demersal fisheries, NE Atlantic

## Abstract

**Simple Summary:**

The silver scabbardfish, *Lepidopus caudatus*, is an important demersal deep-sea species commercially exploited in the Azores archipelago. Portugal has the greatest average landings in the world with Azores being the main contributor. Unfortunately, this species has no specific measurement regulations regarding its fishing. This study aimed to fill some basic information about the species in order to further use it as a foundation for management advice. The objective was to determine the spatial distribution, life history parameters, and abundance changes using scientific survey and fishery-dependent data from 1990 to 2019. The results showed the distribution of the species was mainly associated with areas closer to islands and seamounts (200–400 m) with preference for sandy bottoms. The life history parameters exposed a slow-growing species, with a high predominance of females in the population. The abundance revealed a declining tendency, and the exploitation rate suggested the species could be slightly overexploited. Overall, this resource could be considered as vulnerable, and the parameters calculated in this study could be the basis for future works such as analytical stock assessment, to serve as a guidance for its preservation and sustainable exploitation.

**Abstract:**

Deep-sea fisheries are of important economic value. Therefore, it is necessary to generate biologically and ecologically based fishing plans to make this fishery sustainable over time. The silver scabbardfish, *Lepidopus caudatus*, is a worldwide-distributed demersal fish, commercially exploited in the Azores. Despite *L. caudatus* high landings and discharges in the region, information about its ecology, population structure, biology and fisheries remains little-known. This work analyzed scientific survey and fishery-dependent data from the past 30 years to understand the changes in abundance, spatial distribution and life history of this species. *Lepidopus caudatus* spatial distribution was associated with depths between 200 and 400 m, close to seamounts and islands, and on sandy bottoms. The size structure varied yearly, and the population was dominated by females (sex ratio, M:F = 0.46:1). Growth rates were between the estimated values in previous studies in the Azores and indicated a slow-grower species (L_∞_ = 171.62 cm, k = 0.12 year^−1^, Φ’ = 3.52). The abundance indices highlighted a declining tendency, and this result was backed by the high exploitation rate for the fish in the region (E = 0.53). Lack of management measurements and the species’ vulnerability could lead to the depletion of this species.

## 1. Introduction

Deep-sea fishes, defined as the ones occurring from a 200 m depth [1,2], have been exploited since at least 450 years ago [3]. This activity became industrialized in the 1950s, and nowadays, around 300 species are exploited commercially [4]. The total catch in global fisheries for deep-sea fish species is less than 1% of the total fish marine catch, but for some places such as the Azores archipelago (NE Atlantic), they are an important part of their total regional catch, representing about 60% of the total landed weight [1].

The economic and ecological sustainability of deep-sea fishing resources over time is still debated [5,6]. The low productivity of deep-sea species due to their low fecundity, late maturity, and slow growth, and high level of biomass exploitation make these resources vulnerable to overfishing, as reflected by the rapid depletion of deep-sea orange roughy *Hoplostethus atlanticus* in some stocks of New Zealand [7]. The information provided by biological characteristics and spatial patterns of the stocks are key aspects to generate ecologically and economical meaningful management programs [2,6]. However, the scientific understanding of deep-sea species is very limited, and this affects the efficacy of stock assessment models [2].

Of the top 50 species exploited in deep-sea fisheries, *Lepidopus caudatus* is 24th [4]. *Lepidopus caudatus* is a trichiurid demersal fish distributed in the temperate waters around the Eastern Atlantic Ocean, including Macaronesian archipelagos, the Western Mediterranean Sea, the Southwest and Southeast Pacific Ocean, and the Southern Indian Ocean [8,9]. It occurs over sandy and muddy bottoms along the continental shelf and slopes at depths from 42 m to 620 m [9,10]. The species feeds on small prey such as crustaceans, fishes, and cephalopods [11]. It forms schools and migrates at night to shallower depths and on occasions can be found inshore on upwelling zones of deep water [9].

This species is caught by bottom and pelagic trawlers, and longline fisheries [12]. Globally, the landing tendency of *L. caudatus* from 1950 to 2019 reached a peak of 27,000 tons in the 1990s and rapidly decreased to a minimum of 4000 tons in the early 2000s [13]. After 2001, the landings slowly augmented with a peak in 2014 of 15,000 tons and subsequently diminished again [13]. On average, from 1950 to 2019, the top countries, in order, that exploited the *L. caudatus* were Portugal, South Africa, New Zealand, Italy, and Spain [13].

Portugal’s landings follow the global pattern, with the highest peaks in the 1990s and in the 2010s [13]. Since the 2000s, the Azores landings of *L. caudatus* have gained importance in the Portuguese fisheries and this region was responsible for the Portuguese uplift in the 2010s landings; however, the landings in Azores have been decreasing since then [14]. In the Azores, *L. caudatus* is captured by using hooks and lines and is one of the twenty-two stocks identified as priorities for monitoring and assessment [15]. Economically, for the archipelago, it is ranked eleventh in terms of total landed value considering non-stranding stocks, valued at EUR 0.5 M on average per year [8].

Despite its importance, the scientific knowledge on biological and ecological traits of *L. caudatus* is still limited worldwide. It is known that this species grows larger and faster than other economically important deep-sea fishes, suggesting it could be a possible resource for sustainable fisheries [16]. On the other hand, the Mediterranean Sea has experienced an overfishing scenario for *L. caudatus* in the Strait of Sicily [12]. In the Azores, the real stock status is unknown and previous studies highlighted the need for more information on population structure and dynamics to inform reliable management strategies for the conservation of this species [16].

In this context, this study aimed to explore historical fishery-dependent and independent data to determine the biological and ecological characteristics of *L. caudatus* in the Azorean waters. The studied aspects included spatial distribution, size structure, sex ratio, growth parameters, and mortality rates. The potential effects of fishing in the annual abundance indices were also investigated to facilitate stock assessment and support fishery management measures in the region.

## 2. Materials and Methods

### 2.1. Data Collection

The sources of the data analyzed in this study were derived from scientific surveys, commercial catches, and official commercial landings in the Azores archipelago (ICES Subdivision 27.10.a.2). The Azores archipelago is a Portuguese autonomous region localized in the North Atlantic Ocean. It is composed by nine volcanic islands with little geological continental shelf, and several hundred of seamounts, [17]. This ecoregion is part of a larger open ocean ecosystem and sits astride the Mid-Atlantic ridge [17].

#### 2.1.1. Scientific Surveys

**Figure 1:**
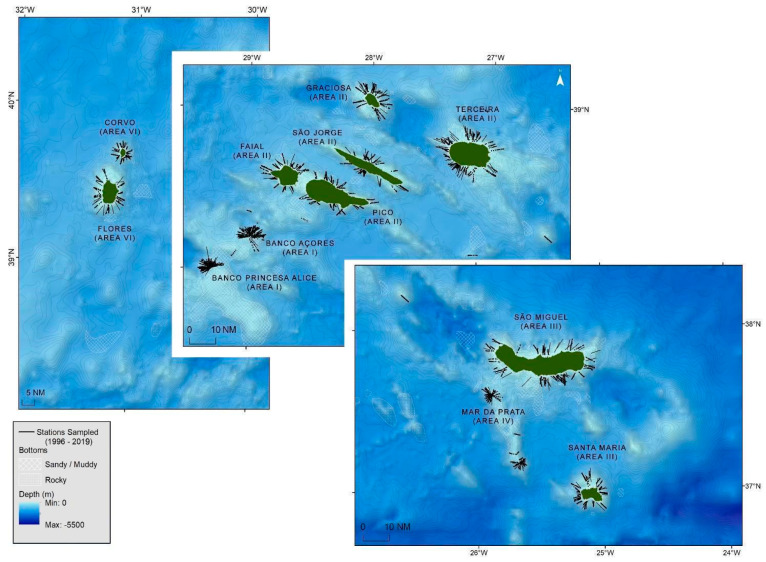
Azores map with bathymetry, substrate type and sampling stations from the scientific survey ARQDAÇO (1996–2019).

From 1996 to 2019, the spring bottom longline survey (ARQDAÇO) was performed and covered the islands (e.g., Corvo, Flores, Graciosa, and Santa Maria) and seamounts (e.g., Banco Açores, Banco Princesa Alice, and Mar da Prata) of the Azores archipelago (Figure 1). The ARQDAÇO followed a stratified random sampling design in which each sampling area was divided into depth strata with 50 m intervals down to 600 m depth [18]. Each bottom longline set was deployed perpendicular to the isobaths (Figure 1). For each caught *L. caudatus* specimen (*n* = 2832), fork length (FL, cm) and sex were recorded. Details on the survey design can be consulted in Pinho et al. [18].

#### 2.1.2. Commercial Catches

Commercial catch data were collected by the vessels’ captains from the local fleet during their landings at Azorean ports from the period of 1990–2017. The structured inquiries (*n* = 31,616) were conducted within the European Commission’s data collection framework [19]. For each inquiry, the vessel ID and size, departure and arrival dates, fishing gear type, average depth zone of the fishing operation, and catch in weight by species were obtained.

#### 2.1.3. Official Commercial Landings

Official landings (in tons) were obtained from the Azores Auction Services (Lotaçor S.A.) for the period 1990–2017. Data recorded for each fishing operation included vessel identification, *métier*, and catch in kg by species. Information on FL for combined sexes was available for a sample (*n* = 29,992) of *L. caudatus* landed from 1990. 

### 2.2. Data Analyses

#### 2.2.1. Spatial Distribution

To understand the distribution of *L. caudatus* in the Azores in relation to environmental variables, a Generalized Additive Model (GAM) was used. GAMs are generalized linear models that can model more complex relationships than linear through link functions [20,21,22,23] and can deal with zero-inflated data (in this study, 91% of zeros) by two-step modeling (i.e., hurdle models) [24]. This approach fits the data in two different models, binomial, which uses the data converted into presence–absence, and the Gaussian, which fits only the positive counts of abundance [24]. Through this, it is possible to identify if the mechanisms producing the presence or absence of the species are different from the ones determining the abundance [25].

The predictive variables were latitude and longitude (as an interaction term), depth, and substrate type. Latitude and longitude as well as absolute depth (0–600 m) were obtained during gear deployment in the ARQDAÇO surveys. The substrate type was extracted from EMODnet seabed habitat compilations (www.emodnet-seabedhabitats.eu, 5 March 2022) and categorized as coarse sediment (C.Sed), mixed sediment (Mix.Sed), mud (Mud), muddy sand (Mud.S), rock (Rock), sand (Sand), or sandy mud (Sand.M).

The used model was:

(1) g(η)= s (longitude,latitude)+ s(depth)+ factor(sediment type)

The η represents the probability of the presence of *L. caudatus* when the binomial response data was used, or the log-transformed abundance when the positive count data was employed. g is the link function between η and each additive predictor: logit link for the binomial GAM and log-link for the Gaussian GAM. s is the thin-plate regression spline fitted for the predictors. Models were fitted using the mgcv package [23], under the R programming environment [26].

#### 2.2.2. Size Structure

Differences in FL composition regarding data origin (survey-derived, *n* = 2832 vs. landing-derived, *n* = 29,992) and surveyed area (seamounts, *n* = 1923 vs. islands, *n* = 909) were investigated by applying a two-sample Kolmogorov–Smirnov (K-S) test using stats R package version 4.3.0 [26]. Differences in the mean FL among depth strata and years were tested through the Welch’s heteroscedastic F test and Games–Howell post hoc correction [27], with SPSS software version 28.0 [28].

#### 2.2.3. Sex Ratio

Proportions of males to females (M:F) from the population, by FL–class, depth stratum and year were compared with the expected 1:1 ratio using the goodness of fit chi-square tests. For this, the scientific survey data were used because fishery-dependent samples were not sexed. The analysis was conducted using stats R package [26].

#### 2.2.4. Growth Parameters

The growth parameters were estimated through the von Bertalanffy growth function (VBGF) [29] using monthly FL frequency data (5 cm class interval) derived from the available official landings (*n* = 6743) for the most recent period 2010–2017. As the FL data were not available for males and females separately, growth parameters were estimated for combined sexes. The original VBGF model was adapted to remove theoretical age at length zero (t_0_):

(2) $$\mathrm{Lt}\;={\;L}_{\infty }\;\left(1\;–e^{-k\left(t\right)}\right)$$

Lt is the fish length (cm) at age t (year), L_∞_ is the asymptotic length (cm), i.e., the length that fish of a population would reach if they grew indefinitely, and k is the growth coefficient that expresses how fast (year^−1^) the asymptotic length is approached [30]. These two parameters and the growth performance index (Φ’), a parameter that enables to compare and determine the reliability of growth parameters [31] were calculated by electronic length–frequency analysis using a bootstrapped method with a genetic algorithm (ELEFAN_GA_boot; [32]) within the TropFishR [33,34] and fishboot [32] packages in R [26]. Bootstrap experiments were based on 1000 resamples. Using data from different studies about *L. caudatus* growth parameters [8,16], the Φ’ values were calculated to compare with the Φ’ obtained in this study. The formula used was [35]:

(3) $$\Phi ’\;=2\log_{10}{L\;}_{\infty }+{\;\log}_{10}k\;$$

#### 2.2.5. Mortality Parameters

Mortality rates were calculated using the FL data taken from the official landings for the period 2010–2017. The total mortality rate (*Z*; year^−1^) was estimated based on the linearized length–converted catch curve [30] within the TropFishR R package [33,34]. The natural mortality (*M*; year^−1^), which is the death of fish in a stock due to predation, disease, or old age [30], was calculated as the average value of natural mortality assessed through different methods using 18 °C as the mean temperature as did Santos et al. [36]. Fishing mortality (*F*; year^−1^), defined as the fish death attributed to fishing activities, was obtained from the relationship [30]:

(4) F=Z – M

The exploitation rate (E), i.e., the fraction of fish caught by fishing against the total number of individuals dead of all the causes [37], was determined by [38]:

(5) E =F/(F+M)

#### 2.2.6. Trends in Abundance

Three abundance indices were constructed to compare mean annual abundances through the different databases: the relative population number (RPN; ind. 10^−3^ hooks) from survey data, landing per unit effort (LPUE; kg landings^−1^ vessel^−1^) from official landings and catch per unit effort (CPUE; kg days at sea^−1^ vessel^−1^) from commercial catches. As RPN data were obtained directly from the scientific survey with a standardized experimental design, the mean annual abundance index for this index was calculated directly and normalized by the min–max year values. Nevertheless, CPUE and LPUE data came from fishery-dependent sources, so these data can produce biased abundances because of differences in vessel sizes, fishing gear, and even target species [39]. Therefore, it is necessary to standardize these data by removing the impact of the different factors that affect the catches. A hurdle–lognormal Generalized Linear Model (GLM) was performed to determine which characteristics of the fishing-related catches had impacts on the yearly abundance indices [39]. The possible factors affecting the LPUE were year, quarter, vessel length, *métier*, target effect (percentage of the capture of *L. caudatus* in relation to the total), and for CPUE were year, quarter, vessel length, gear type, depth, target effect (See Table S1 for details in the levels of each factor). Statistical details about the standardization procedure are found in Santos et al. [39]. The GLM was run using the lsmeans R package [40]. The significance levels of all statistical analyses were set at a *p*-value < 0.05.

To compare the general LPUE, CPUE, and RPN trends, linear models were applied to the annual standardized catch rates using the R function lm on the ggplot R package [41]. To know whether the three linear models were distinct, differences in direction or magnitude (i.e., differences in slopes or intercepts) were investigated. Analysis of Covariance (ANCOVA) was performed to look for parallelism in the slopes of the linear models [42] by testing the significance (*p* < 0.05) of the interaction of the covariate (year) and the predictive variable (abundance index) [43,44] using car package [45] in R software [26]. A post hoc Tukey test was used to determine which slopes differed in magnitude (i.e., had different intercepts) [43,44] with the multcomp package [46] also in R software [26].

## 3. Results

### 3.1. Spatial Distribution

**Figure 2:**
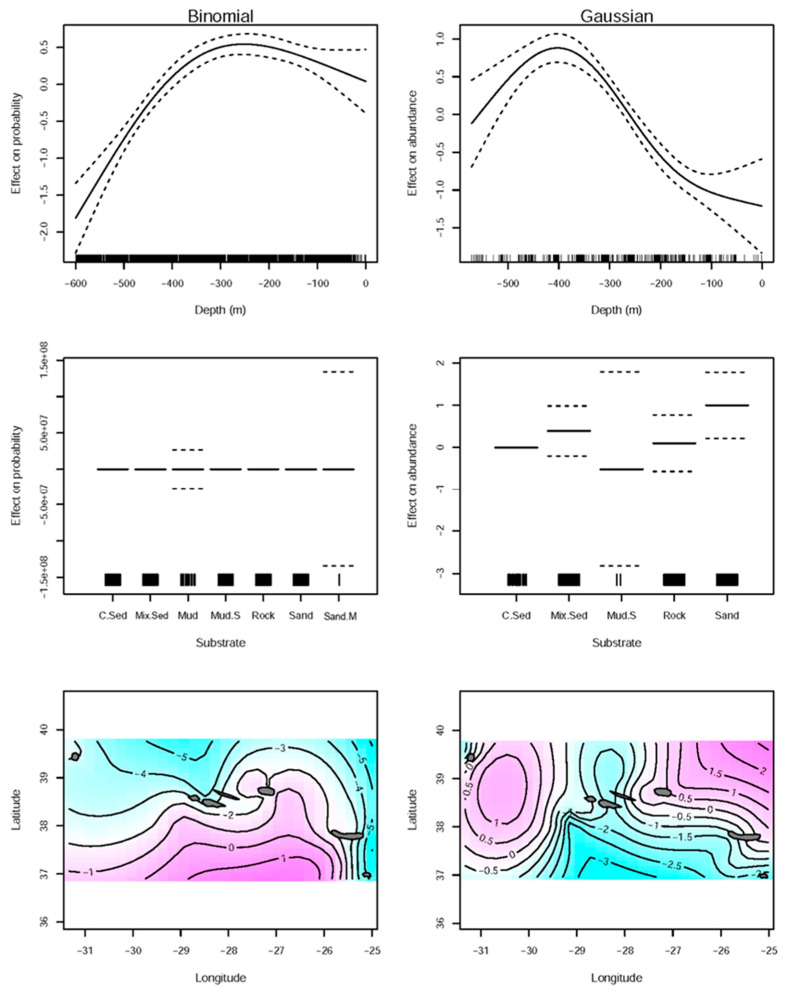
Residual plots of the binomial (the left column of plots) and Gaussian (right column of the plots) generalized additive models (GAMs) developed to predict and determine the effect of the environmental variables in the spatial distribution (presence–absence and abundance) of *Lepidopus caudatus* in the Azorean waters. In the plots of depth and substrate type, each mark along the x-axis corresponds to a single observation and dashed lines correspond to the 95% confidence intervals. The purple color from the longitude and latitude plot represents a higher probability of occurrence or abundance in determined latitude and longitude, and the blue color indicates less probability or less abundance of individuals.

The GAM for the effect of substrate type, depth, longitude and latitude in the spatial distribution of *L. caudatus* in the Azores explained 14.9% of the deviance for the presence of the fish (binomial) and 38% for the abundance (Gaussian) (Table 1). The substrate types were not important in general, the only significant (*p* < 0.05) was sand substrate as a predictor of higher abundances of the fish (Table 1, Figure 2). The smooth terms of depth together with longitude and latitude were relevant variables for the presence and abundance of *L. caudatus* model’s fit (*p* < 0.05) (Table 1). The highest abundances and probabilities of presence were found in depths between 200 and 400 m (Figure 2). Geographically, the probability of the presence of *L. caudatus* was higher in the southern regions of the central islands and closer to the islands, and the abundance was higher in the western and eastern parts.

### 3.2. Size Structure

**Figure 3:**
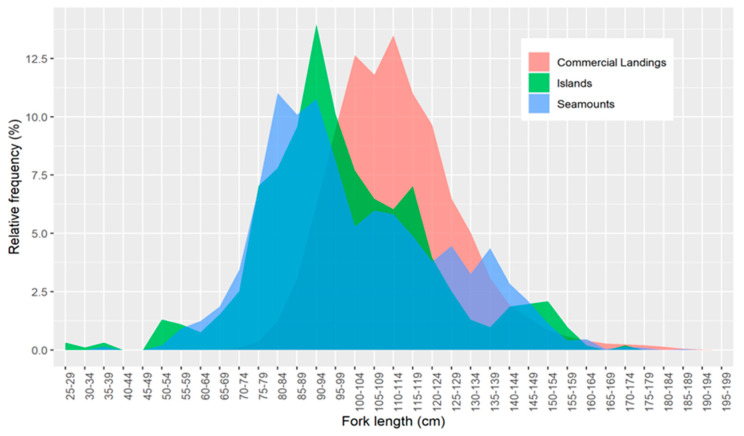
Relative frequency of fork length class (FL) (5 cm interval) of *Lepidopus caudatus* in Azorean waters, derived from the scientific survey (around the islands and seamounts) and fishery-dependent data. Commercial landings *n* = 29,991; Seamounts *n* = 1923; Islands *n* = 909.

The FL of *L. caudatus* in the Azorean waters ranged from 25 cm to 198 cm (Figure 3). No significant differences in the length frequency distribution were found between seamounts and islands (K-S test; D = 0. 0862, *p*-value = 0.538, Figure 3). On the other hand, both survey-related size structures (seamounts and islands) were significantly different from the commercial landings (seamounts vs. commercial landings: K-S test; D = 0.155, *p*-value = 0.0303; islands vs. commercial landings: D = 0.184, *p*-value = 0.00556, Figure 3).

**Figure 4:**
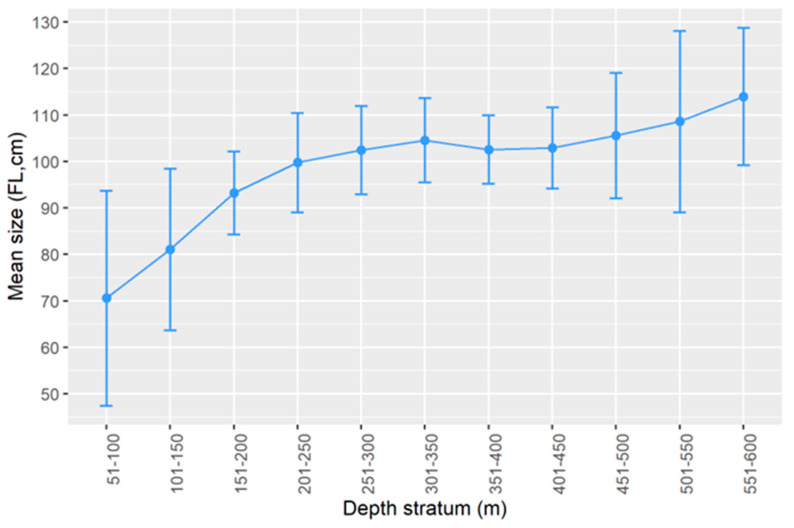
Mean fork length (FL) by depth stratum of *Lepidopus caudatus* in the Azorean waters from scientific surveys (1996–2019). The bars represent the 95% confidence intervals.

*Lepidopus caudatus* showed differences in sizes regarding the depth (Welch test; F = 28.515, *p* < 0.001) with a bigger–deeper trend in the Azores (Figure 4).

**Figure 5:**
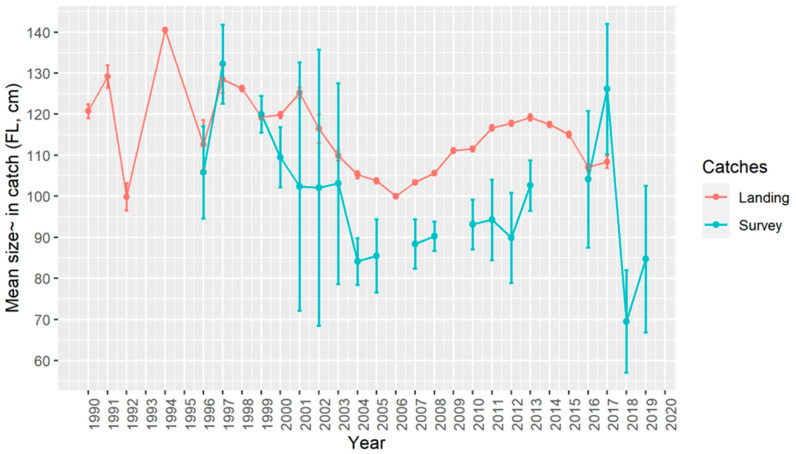
Annual mean fork length (FL) of *Lepidopus caudatus* from the Azores derived from official commercial landings (1990–2017) and scientific surveys (1996–2019). The bars represent the 0.95 confidence intervals.

The changes in the mean sizes were similar both in official landings and surveys across the years (Figure 5). During the late 1990s and early 2000s, the mean sizes were bigger but steeply diminished until reaching a valley around the year 2005. The mean sizes were different by year for official landings (Welch test; F = 336, *p* < 0.05) as well as for surveys (Welch test; F = 97.8, *p* < 0.05). The Games–Howell test for differences by pairs of years in landings means size catches, showed most of the combinations of years had significant differences (Table S2). The differences by pairs of years in the survey mean size catches showed a pattern of more significant differences in mean sizes between pairs of years the farther the two years were (Table S3).

### 3.3. Sex Ratio

**Figure 6:**
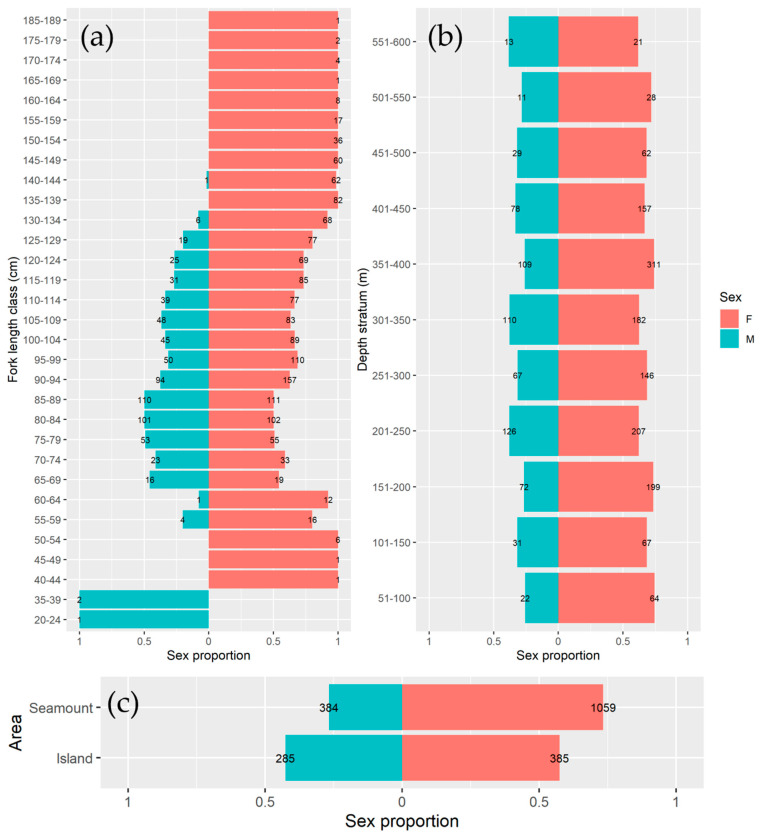
Sex proportion of *Lepidopus caudatus* in the Azorean waters derived from scientific survey by (**a**) fork length class (FL, cm) (**b**) depth stratum (m) and (**c**) surveyed areas (seamounts vs. islands). The number on the top of each column represents the number of individuals (n). F represents females and M males.

The goodness of fit chi-square test confirmed that the ratio M:F (0.46:1) did not correspond to an equal sex ratio (chi-squared = 284.25, *p* < 0.05) and therefore, it is biased towards females. The sex ratio by length class demonstrated that females dominated the largest size classes (Figure 6a, Table S4). By depth, females were the dominant group in all strata, except the stratum 551–600 m (Figure 6b, Table S5). The predominance of females was observed in islands and seamounts regions (Figure 6c, Table S6).

### 3.4. Growth Parameters

**Figure 7:**
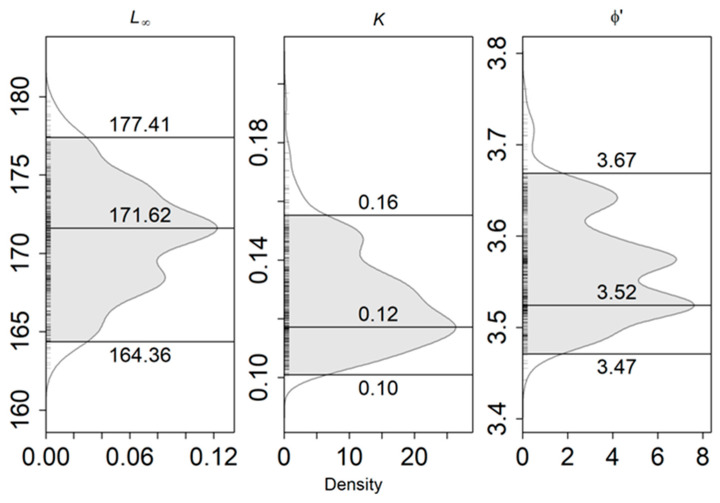
Growth parameters’ estimates for pooled sexes, with 0.95 confidence intervals for *Lepidopus caudatus* in the Azorean waters from official landings data (2010–2017). L_∞_ represents the asymptotic length (cm), k is the growth coefficient (year^−1^) and Φ’ is the growth performance index.

The size classes used in ELEFAN_GA ranged from 84 to 173 cm. The estimated growth parameters and their respective 0.95 confidence intervals for *L. caudatus* are presented in Figure 7. The asymptotic length (L_∞_) was 171.62 cm, the growth coefficient (k) was 0.12 year^−1^ and the growth performance index (Φ’) was 3.52.

### 3.5. Mortality Rates

**Figure 8:**
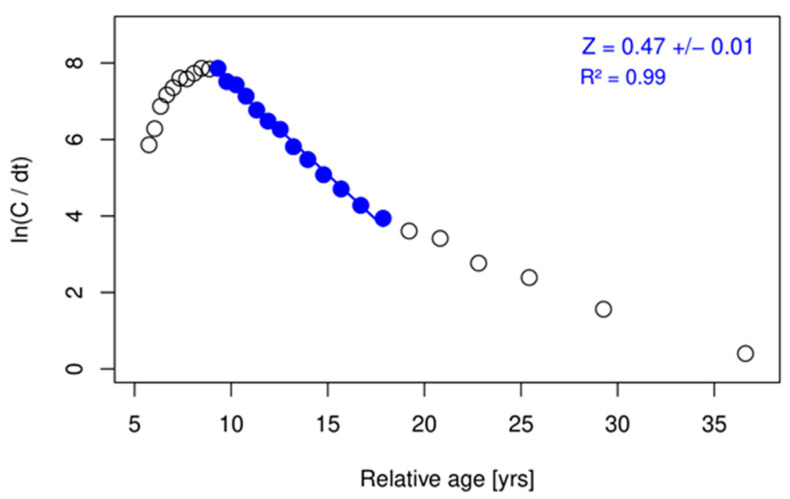
Estimate of total mortality (*Z*) with the linearized length-converted catch curve for *Lepidopus caudatus* in the Azores using data for pooled sexes from official landings (2010–2017).

Total mortality (*Z*), fishing mortality (*F*), and natural mortality (*M*) for the period 2010–2017 were estimated at 0.47 ± 0.01 year^−1^, 0.25 year^−1^, and 0.22 ± 0.03 year^−1^, respectively. The exploitation rate (E) was determined at 0.53. Details on the estimated *Z* and *M* values are shown in Figure 8 and Table 2, respectively.

### 3.6. Trends in Abundance and Catch

**Figure 9:**
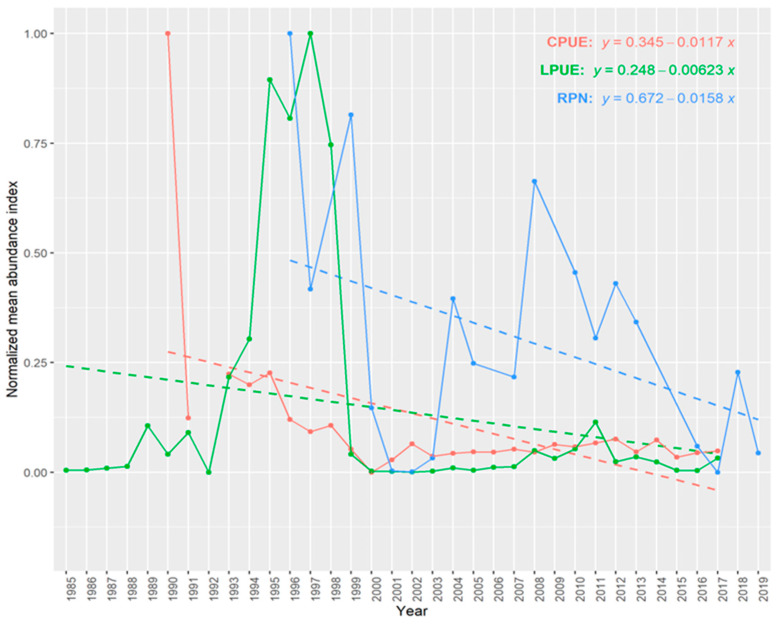
Min–max normalized mean abundance indices for different years of *Lepidopus caudatus* in the Azores. The abundance indices used were standardized CPUE (kg days at sea ^−1^ vessel ^−1^), LPUE (kg landing^−1^ vessel^−1^) and survey-derived RPN (ind. 10^−3^ hooks). The dashed lines represent the linear regressions for each abundance index.

The RPN abundance index showed interannual variability (Welch’s test; F = 7.745, *p* < 0.001). The three normalized mean abundance indices (RPN, CPUE and LPUE) showed two peaks in abundance in the mid-1990s and mid-2000s (Figure 9). Nevertheless, the linear models illustrated an overall declining tendency of the abundance of *L. caudatus* over the years. The ANCOVA test resulted in a non-significant difference in the interaction term (F = 0.657, *p* = 0.521; Table 3), indicating that the slopes of the three indices behave similarly, declining. The post hoc Tukey test determined that the RPN intersect was different from the CPUE and LPUE’s (t = 3.080, *p* = 0.008; t = 3.073, *p* = 0.008). This is an indication that CPUE and LPUE indices predicted similar abundance values, whereas RPN predicted higher abundances.

## 4. Discussion

This study provides new information on life history aspects and habitat preferences for *L. caudatus*. Like other demersal fishes from the Azorean region [48,49], as well in the Mediterranean [50] and the Atlantic [51], *L. caudatus* showed a spatial distribution mainly predicted by depth. Changes in depth generate variations in the physical and biological environment such as light intensity, temperature, salinity, predation and competition [50,52,53]. These variations, create vertical gradients [53] possibly establishing different niches for the species, thus affecting the species distribution. *Lepidopus caudatus* in the Azores was mostly present between 200 and 400 m, as in other parts of the world [9,10,11,16,54,55].

The presence of *L. caudatus* in the Azores was linked to the southern parts of the archipelago and the abundance to the western and eastern regions. These patterns can be related to the depths and substrate types found in those geographical areas. In the southern and western areas to the central islands group, several seamounts with depths relevant to the distribution of the fish, up to 500 m, can be found (i.e., Princess Alice and Azores Banks in the south, and Mid-Atlantic Ridge seamounts in the west) [17]. Additionally, the eastern regions, close to Terceira and São Miguel, have areas with sandy bottoms. In the generalized additive model, sandy bottoms were the only important substrate type to predict the distribution of *L. caudatus* coinciding with what was stated previously in the literature [9]. Parra et al. [52] found that substrate type was an influential variable to explain demersal fish distribution in the Azores. Considering that these habitats characteristics (less than 600 m) correspond to less than 1% of the Azores Economic Exclusive Zone, the limited availability of habitat could be a critical condition that makes these animals vulnerable in the Azores.

The maximum FL found in this study (198 cm) was bigger than the previously reported for the Azores archipelago (194 cm) and other regions, such as 196 cm in the Mediterranean Sea [8]. The differences in size between commercial landings and scientific surveys are probably associated with size-selective fishing [16,18]. The observed bigger-deeper trend is a common feature for demersal fishes and could be attributed to bathymetric differences in food availability and avoidance of inter- and intra-specific competition and predation [53]. Yearly variations in mean sizes were observed, because after big sizes were exploited, small sizes landed in greater proportions (e.g., the year 2006). This pattern may be explained by the “fishing down” hypothesis [56,57], which dictates that fishing intensification produce a change in size within a population [57]. A process that could elucidate the reason behind this hypothesis, could be the historical removal of larger individuals by the fishery [58]. This pattern has been observed in other parts of the world with several species [59,60].

The prevalence of females in the population observed in this study coincides with previous works made in the Azores [16,61], and the Mediterranean [54]. This is a common tendency in deep-sea fauna [62]. Several reasons could explain this difference. From a biological and ecological point of view, there could exist a real unequal sex ratio in the population [16,63], as the males could have higher mortality rates than the females [63] or as an evolutionary trade to favor oocyte production to maximize reproductive effort [62]. Additionally, the sex bias could be a sampling artifact due to the fishing gear [63]. Female and males could have different behavior in front of bait; for example, some female fish species could spend more time feeding than males [63]. Further studies are, therefore, encouraged to clarify which factors are responsible for the prevalence of females in the Azorean population. Females of *L. caudatus* were also dominant in the largest size classes as noted in previous studies in the Azores and Mediterranean [54,61], and this could be related to fast-growing and long-living trends in females [54]. The presence of more females in the seamounts than in the islands can be explained because of more suitable habitat for females as the seamounts have deeper bathymetry than coastal areas.

There is evidence that the *L. caudatus* stock from the Mediterranean Sea is overexploited [12], and the fisheries’ intensity affects the population size and, consequently, it reduces the L_∞_, and accelerates k [30]. Growth parameters were consistent with previous estimates in the Azores based on direct readings of otoliths [16], although lower than the Mediterranean Sea [11,55] and higher than New Zealand [64], confirming that *L. caudatus* is a long-living and slow-growing. Besides that, the estimates generated from ELEFAN routines are robust to changes in size structure due to fishing pressure [65].

Bigger abundances of *L. caudatus* were followed by population drops. Both density-dependent and independent processes may interact to shape the population abundance [66]. Figueiredo et al. [16] found variations in the abundance of this species in the Azores between the years 2004–2010 and hypothesized a possible fishing pressure causality. They observed relation between high peaks in abundance of *L. caudatus* and low peaks of blackspot seabream *Pagellus bogaraveo*, the most important commercial demersal species in the Azores. However, when we compared the landings from the blackspot seabream reported from 1985 to 2018 [67] with *L. caudatus* abundance index, there was no such pattern.

The three-abundance index indicated a decline in abundance over the years for this species in the Azores. This coincides with the abundance reduction in important commercial species in this region in the same period [67]. Furthermore, the scientific survey predicted different and higher abundance than fishing-dependent indices. This could be explained by the fact of different fishing grounds of the scientific survey and the commercial fisheries’ activities. The scientific surveys take place in areas closer to the islands and main seamounts, which have historically been most affected by the fishing activities. However, since the year 2002, fishing effort in these areas has decreased due to a fishing ban imposed by the Azorean government to avoid overexploitation of the resources [68].

In addition to the abundance decline of *L. caudatus* in the Azores, the exploitation rate (E) estimated for the most recent period (2010–2017) was slightly above the optimum level of 0.5, and the fishing mortality (*F*) was higher than natural mortality (*M*), indicating that this species is currently intensively exploited [38]. Nowadays, there are no specific regulation measurements for this species (e.g., total allowable catch, minimum landing size or moratorium during spawning season) [8] even though the discards are high, and Portugal has led this fish’s exploitation worldwide. The information gathered in this study could be used for further stock assessment analysis which could contribute to the management of this species.

## 5. Conclusions

Valuable information necessary for the stock assessment and management of *L. caudatus* in the Azores archipelago was determined in this research, such as the spatial distribution, size structure, growth parameters, mortality rates, sex ratio and trends in abundance and catches. These results suggest an intensively exploited fishery in the Azores, sustaining the decline in abundance for the species. Even though the stock identity for this species is undetermined in the archipelago, we could suggest it is a different stock from that of the Mediterranean Sea and Canary Islands, due to differences in the growth parameters. To determine if there are different populations of *L. caudatus* in the Atlantic and the Mediterranean is vital for management of the resource, but further studies for connectivity, such as larval ecology and genetics, are needed for proper stock characterization. Given the fact that Azores is the top region of Portugal exploiting this resource and the vulnerability of the *L. caudatus* as a slow-grower species, it is vital to generate measurements of protection for this species in the Azores. Future work should focus on assessing the stock size, reproductive aspects of this animal, and biological reference points with data-limited methods to produce the maximum sustainable yield (MSY) for *L. caudatus*.

## Figures and Tables

**Table 1:** Detailed summary table of generalized additive model (GAM) results for *Lepidopus caudatus* abundances and presence, derived from scientific surveys (1996–2019) in the Azores. Substrate type: coarse sediment (C. Sed), mixed sediment (Mix.Sed), mud (Mud), muddy sand (Mud.S), rock (Rock), sand (Sand), and sandy mud (Sand.M). The significative codes are: 0 ‘***’ 0.01 ‘*’ 0.05 ‘.’ 0.1 ‘ ’ 1.

Family	Link Function		Formula				Adjusted R^2^		Deviance Explained
Binomial	logit		RPN.Bi ~ s (longitude, latitude) + s(depth, k = 4) + substrate				0.0963		14.9%
Gaussian	identity		RPN ~ s (longitude, latitude) + s(depth, k = 4) + substrate				0.347		38%
Binomial					Gaussian				
Parametric coefficients									
	Estimate	Std. Error	z Value	Pr (>|z|)		Estimate	Std. Error	z Value	Pr (>|z|)
(Intercept)	−2.909 × 10^0^	2.414 × 10^−1^	−12.049	<2 × 10^16^ ***	(Intercept)	−1.6603	0.2943	−5.642	2.8 × 10^−8^ ***
SubstrateMix.Sed	2.087 × 10^−2^	2.425 × 10^−1^	0.086	0.931	SubstrateMix.Sed	0.3895	0.2963	1.315	0.1892
SubstrateMud	−3.901 × 10^1^	1.342 × 10^7^	0.000	1.000	Substrate Mud.S	−0.5146	1.1527	−0.446	0.6555
Substrate Mud.S	3.360 × 10^−1^	8.086 × 10^−1^	0.415	0.678	Substrate Rock	0.1021	0.3341	0.306	0.7601
Substrate Rock	9.064 × 10^−2^	2.636 × 10^−1^	0.344	0.731	Substrate Sand	0.9959	0.3911	2.546	0.0112 *
Substrate Sand	−1.124 × 10^−1^	2.983 × 10^−1^	−0.377	0.706					
Substrate Sand.M	−3.394 × 10^1^	6.711 × 10^7^	0.000	1.000					
Approximate significant smooth terms									
	edf	Ref. df	F	*p*-Value		edf	Ref. df	F	*p*-Value
s(longitude, latitude)	22.660	25.956	269.82	<2 × 10^−16^ ***	s(longitude, latitude)	20.630	24.713	6.412	<2 × 10^−16^ ***
s(depth)	2.688	2.929	90.51	<2 × 10^−16^ ***	s(depth)	2.926	2.995	37.265	<2 × 10^−16^ ***

**Table 2:** Estimates of natural mortality (*M*; year^−1^) for *Lepidopus caudatus* in the Azores, estimated from the empirical relationships between the estimated asymptotic length (L_∞_; cm) and growth rate coefficient (k; year^−1^).

Reference	Equation	Result
Beverton and Holt (1959)	$M=5/t_{max}$	0.22
Taylor (1960)	$M=2.996/t_{max}$	0.13
Tanaka (1960)	$M=3/t_{max}$	0.13
Alverson and Carney (1975)	$M=3k/\left(exp\left(0.38t_{max}*k-1\right)\right)$	0.34
Pauly (1980)	M=Exp(−0.0066−0.279Log(Linf)+0.6543Log(k)+0.4634Log(T))	0.53
Hoening (1983)	$M=3/t_{max}$	0.13
Alagajara (1984)	$M=4.6/t_{max}$	0.20
Djabali (1993)	M=1.0661Linf^−0.1172∗k^0.5092	0.21
Pauly and Binohlan (1996)	M=−0.1778+3.1687k	0.23
Jensen (1996)	M=1.6k	0.21
Jensen (1996)	M=1.5k	0.20
Cubillos et al. (1999)	M=1.4k	0.18
Frisk (2001)	$M=4.22/t_{max}$	0.18
Hewitt and Hoening (2005)	$M=4.22/t_{max}$	0.18
Mean		0.22

Note: tmax—the approximate maximum age (years) = 3/k [47]; T—water temperature (°C).

**Table 3:** ANCOVA results of differences in the direction of the linear models for the abundance indices. The covariate year interaction with the factorial variable abundance index represents the categories of CPUE, LPUE, and RPN. Signif. codes: 0.001 ‘**’ 0.01 ‘*’.

Mean Abundance ~ Year + Abundance Index + Year * Abundance Index				
	Sum Sq	Df	F Value	Pr(>F)
(Intercept)	0.437	1	7.41	0.008 **
Year	0.231	1	3.91	0.052
Abundance index	0.242	2	2.06	0.135
Year:Abundance index	0.077	2	0.66	0.521
Residuals	4.300	73		

## Data Availability

Data supporting reported results are available from the corresponding author upon reasonable request.

## Supplementary Materials

The following supporting information can be downloaded at: https://www.mdpi.com/article/10.3390/biology11111619/s1, Table S1. Explanatory variables (main factors) used in the model formulations for standardized landing per unit effort (LPUE) and catch per unit effort (CPUE) catch rates; Table S2. Games–Howell multiple comparisons of fork length (FL, cm) from annual landings data; Table S3. Games–Howell multiple comparisons of fork length (FL, cm) from annual scientific survey data; Table S4. Chi-squared test for given probabilities for sex ratio (M:F) at each fork length class; Table S5. Chi-squared test for given probabilities for sex ratio (M:F) at each depth stratum; Table S6. Chi-squared test for given probabilities for sex ratio (M:F) at each surveyed area.
