# Supplementary material for: Life History and Fishing Aspects of the Deep-Sea Silver Scabbardfish Lepidopus caudatus in the Azores
Source: Biology (Basel). 2022 Nov 6;11(11):1619. doi: 10.3390/biology11111619 (PMC9688029; doi:10.3390/biology11111619)
Supplement: Supplementary file 1 [file biology-11-01619-s001.zip › biology-2003393-supplementary.pdf]

Life History and Fishing Aspects of the Deep-Sea Silver Scabbardfish *Lepidopus caudatus* in the Azores

Gloria Mariño-Briceño [1,*], Wendell Medeiros-Leal [1,2], Ualerson Iran Peixoto [1,2], Mário Pinho [1,2] and Régis Santos [1,2]

[1] Okeanos–UAc Instituto de Investigação em Ciências do Mar, Universidade dos Açores, Rua Prof. Dr. Frederico Machado, 4, 9900-138 Horta, Portugal
[2] IMAR Instituto do Mar, Departamento de Oceanografia e Pescas, Universidade dos Açores, Rua Prof. Dr. Frederico Machado, 4, 9901-862 Horta, Portugal
* Correspondence: gloritam94@gmail.com; Tel.: +351-292200400

Supplementary material

Table S1. Explanatory variables (main factors) used in the model formulations for standardized landing per unit effort (LPUE) and catch per unit effort (CPUE) catch rates.

Table S2. Games–Howell multiple comparisons of fork length (FL, cm) from annual landings data.

Table S3. Games–Howell multiple comparisons of fork length (FL, cm) from annual scientific survey data.

Table S4. Chi-squared test for given probabilities for sex ratio (M:F) at each fork length class.

Table S5. Chi-squared test for given probabilities for sex ratio (M:F) at each depth stratum.

Table S6. Chi-squared test for given probabilities for sex ratio (M:F) at each surveyed area.

Table S1. Explanatory variables (main factors) used in the model formulations for standardized landing per unit effort (LPUE) and catch per unit effort (CPUE) catch rates.

LPUE			CPUE		
**Variable**	**Type**	**Observations**	**Variable**	**Type**	**Observations**
Year	Categorical (33)	Period: 1985-2017	Year	Categorical (27)	Period: 1990-2017
Quarter	Categorical (4)	1: January-March<br>2: April-June<br>3: July-September<br>4: October-December	Quarter	Categorical (4)	1: January-March<br>2: April-June<br>3: July-September<br>4: October-December
Vessel length	Categorical (5)	1: ≤ 10 m<br>2: > 10 and ≤ 12 m<br>3: > 12 and ≤ 18 m<br>4: > 18 and ≤ 24 m<br>5: > 24 and ≤ 40 m	Vessel length	Categorical (5)	1: ≤ 10 m<br>2: > 10 and ≤ 12 m<br>3: > 12 and ≤ 18 m<br>4: > 18 and ≤ 24 m<br>5: > 24 and ≤ 40 m
Métier	Categorical (11)	HDP: hand picking<br>HUN: species removal by hunting<br>FPO_CRU: pots and traps for crustaceans<br>GNS_FIF: gillnets for coastal demersal and pelagic fish<br>LHP_CEP: handlines for cephalopods - squids	Gear	Categorical (5)	LL: Longlines<br>HL: Handlines<br>NT: Nets<br>TP: Traps and pots<br>MG: Multigear
		LHP_FIF: handlines for demersal fish<br>LHP_MDP: handlines locally called "corrico" for pelagic fish<br>LHP_LPF (pole and lines for pelagic fish)	Depth (mean depth of fishing operation)	Categorical (3)	1: Shallow (< 200 m)<br>2: Intermediate (200-600 m)<br>3: Deep (> 600 m)
		LLD: drifting longlines for pelagic and demersal fish<br>LLS_DEF: set longlines for pelagic and demersal fish<br>PS_SPF: purse seines for small pelagic fish	Target effect (percentage of species-specific catch related to the total catch)	Categorical (4)	1: 1st quartile (≤ 25%)<br>2: 2nd quartile (> 25% and ≤ 50%)<br>3: 3rd quartile (> 50% and ≤ 75%)
Target effect (percentage of species-specific catch related to the total catch)	Categorical (4)	1: 1st quartile (≤ 25%)<br>2: 2nd quartile (> 25% and ≤ 50%)<br>3: 3rd quartile (> 50% and ≤ 75%)			4: 4th quartile (> 75%)

Table S2. Games-Howell multiple comparisons of fork length (FL, cm) from annual landings data.

Year (I)	Year (J)	Mean differences (I-J)	Standard Error	Sig.	Lower 95% confidence interval	Upper 95% confidence interval
1990	1991	-8.391*	1.674	<.001	-14.6	-2.18
	1992	20.915*	2.035	<.001	11.37	30.46
	1996	8.19	3.386	0.718	-7.79	24.17
	1997	-7.701*	1.907	0.017	-14.79	-0.61
	1998	-5.473*	0.959	<.001	-9	-1.95
	1999	1.48	0.93	0.997	-1.94	4.9
	2000	0.982	0.977	1	-2.61	4.57
	2001	-4.378*	1.113	0.02	-8.46	-0.29
	2002	4.343	1.99	0.885	-3.08	11.77
	2003	10.901*	1.093	<.001	6.89	14.91
	2004	15.516*	0.995	<.001	11.86	19.17
	2005	16.995*	0.948	<.001	13.51	20.48
	2006	20.758*	0.936	<.001	17.32	24.2
	2007	17.356*	0.937	<.001	13.92	20.8
	2008	15.134*	0.938	<.001	11.69	18.58
	2009	9.645*	0.942	<.001	6.19	13.1
	2010	9.234*	0.938	<.001	5.79	12.68
	2011	4.111*	0.96	0.005	0.58	7.64
	2012	3.035	0.949	0.198	-0.45	6.52
	2013	1.55	0.98	0.998	-2.05	5.15
	2014	3.313	0.949	0.089	-0.17	6.8
	2015	5.689*	0.966	<.001	2.14	9.24
	2016	13.761*	1.006	<.001	10.07	17.45
	2017	12.372*	1.172	<.001	8.07	16.67
1991	1990	8.391*	1.674	<.001	2.18	14.6
	1992	29.306*	2.318	<.001	19.41	39.2
	1996	16.581*	3.563	0.04	0.49	32.67
	1997	0.69	2.206	1	-7.49	8.87
	1998	2.918	1.466	0.951	-2.57	8.4
	1999	9.872*	1.448	<.001	4.45	15.29
	2000	9.373*	1.478	<.001	3.85	14.9
	2001	4.014	1.571	0.653	-1.83	9.86
	2002	12.734*	2.278	<.001	4.27	21.19
	2003	19.292*	1.557	<.001	13.49	25.09
	2004	23.907*	1.49	<.001	18.34	29.47
	2005	25.387*	1.459	<.001	19.93	30.85
	2006	29.149*	1.451	<.001	23.72	34.58
	2007	25.748*	1.451	<.001	20.31	31.18
	2008	23.525*	1.453	<.001	18.09	28.96

	2009	18.036*	1.455	<.001	12.59	23.48
	2010	17.626*	1.453	<.001	12.19	23.06
	2011	12.502*	1.467	<.001	7.02	17.99
	2012	11.426*	1.459	<.001	5.96	16.89
	2013	9.941*	1.48	<.001	4.41	15.47
	2014	11.704*	1.459	<.001	6.24	17.17
	2015	14.081*	1.47	<.001	8.58	19.58
	2016	22.152*	1.497	<.001	16.56	27.74
	2017	20.763*	1.613	<.001	14.77	26.76
1992	1990	-20.915*	2.035	<.001	-30.46	-11.37
	1991	-29.306*	2.318	<.001	-39.2	-19.41
	1996	-12.725	3.746	0.246	-29.37	3.92
	1997	-28.616*	2.491	<.001	-38.89	-18.34
	1998	-26.388*	1.868	<.001	-36.01	-16.76
	1999	-19.435*	1.853	<.001	-29.08	-9.79
	2000	-19.933*	1.877	<.001	-29.54	-10.32
	2001	-25.293*	1.951	<.001	-34.84	-15.75
	2002	-16.572*	2.555	<.001	-27.01	-6.13
	2003	-10.014*	1.94	0.038	-19.57	-0.46
	2004	-5.399	1.886	0.508	-15	4.2
	2005	-3.92	1.862	0.846	-13.55	5.71
	2006	-0.157	1.856	1	-9.8	9.48
	2007	-3.559	1.856	0.908	-13.2	6.08
	2008	-5.781	1.857	0.41	-15.42	3.86
	2009	-11.270*	1.859	0.021	-20.91	-1.63
	2010	-11.681*	1.857	0.017	-21.32	-2.04
	2011	-16.804*	1.868	0.002	-26.43	-7.18
	2012	-17.880*	1.862	0.001	-27.51	-8.25
	2013	-19.365*	1.879	<.001	-28.97	-9.76
	2014	-17.602*	1.862	0.001	-27.23	-7.97
	2015	-15.226*	1.871	0.003	-24.84	-5.61
	2016	-7.154	1.892	0.203	-16.75	2.44
	2017	-8.543	1.985	0.094	-18.08	0.99
1996	1990	-8.19	3.386	0.718	-24.17	7.79
	1991	-16.581*	3.563	0.04	-32.67	-0.49
	1992	12.725	3.746	0.246	-3.92	29.37
	1997	-15.891	3.678	0.058	-32.12	0.33
	1998	-13.663	3.288	0.118	-29.65	2.32
	1999	-6.71	3.28	0.876	-22.7	9.28
	2000	-7.208	3.293	0.821	-23.19	8.77
	2001	-12.568	3.336	0.181	-28.54	3.41
	2002	-3.847	3.722	1	-20.14	12.44
	2003	2.711	3.329	1	-13.26	18.69
	2004	7.326	3.298	0.808	-8.65	23.31
	2005	8.805	3.285	0.587	-7.18	24.79

	2006	12.568	3.281	0.175	-3.42	28.55
	2007	9.166	3.281	0.532	-6.82	25.15
	2008	6.944	3.282	0.85	-9.04	22.93
	2009	1.455	3.283	1	-14.53	17.44
	2010	1.044	3.282	1	-14.94	17.03
	2011	-4.079	3.288	0.999	-20.06	11.9
	2012	-5.155	3.285	0.983	-21.14	10.83
	2013	-6.64	3.294	0.887	-22.62	9.34
	2014	-4.877	3.285	0.99	-20.86	11.11
	2015	-2.501	3.29	1	-18.48	13.48
	2016	5.571	3.302	0.968	-10.41	21.55
	2017	4.182	3.356	0.999	-11.79	20.16
1997	1990	7.701*	1.907	0.017	0.61	14.79
	1991	-0.69	2.206	1	-8.87	7.49
	1992	28.616*	2.491	<.001	18.34	38.89
	1996	15.891	3.678	0.058	-0.33	32.12
	1998	2.228	1.727	1	-4.24	8.7
	1999	9.181*	1.711	<.001	2.77	15.6
	2000	8.683*	1.737	<.001	2.18	15.19
	2001	3.323	1.817	0.981	-3.45	10.1
	2002	12.044*	2.454	<.001	2.94	21.15
	2003	18.602*	1.805	<.001	11.87	25.34
	2004	23.217*	1.747	<.001	16.68	29.75
	2005	24.696*	1.721	<.001	18.25	31.14
	2006	28.459*	1.714	<.001	22.03	34.88
	2007	25.057*	1.715	<.001	18.63	31.48
	2008	22.835*	1.716	<.001	16.41	29.26
	2009	17.346*	1.717	<.001	10.91	23.78
	2010	16.935*	1.716	<.001	10.51	23.36
	2011	11.812*	1.728	<.001	5.34	18.28
	2012	10.736*	1.721	<.001	4.29	17.18
	2013	9.251*	1.739	<.001	2.74	15.76
	2014	11.014*	1.721	<.001	4.57	17.46
	2015	13.390*	1.731	<.001	6.91	19.87
	2016	21.462*	1.754	<.001	14.9	28.02
	2017	20.073*	1.854	<.001	13.17	26.98
1998	1990	5.473*	0.959	<.001	1.95	9
	1991	-2.918	1.466	0.951	-8.4	2.57
	1992	26.388*	1.868	<.001	16.76	36.01
	1996	13.663	3.288	0.118	-2.32	29.65
	1997	-2.228	1.727	1	-8.7	4.24
	1999	6.954*	0.46	<.001	5.27	8.64
	2000	6.455*	0.549	<.001	4.44	8.47
	2001	1.095	0.765	0.999	-1.71	3.9
	2002	9.816*	1.818	<.001	2.98	16.65

	2003	16.374*	0.736	<.001	13.67	19.07
	2004	20.989*	0.58	<.001	18.87	23.11
	2005	22.468*	0.495	<.001	20.66	24.28
	2006	26.231*	0.472	<.001	24.5	27.96
	2007	22.830*	0.473	<.001	21.1	24.56
	2008	20.607*	0.476	<.001	18.86	22.35
	2009	15.118*	0.483	<.001	13.35	16.89
	2010	14.708*	0.476	<.001	12.96	16.45
	2011	9.584*	0.518	<.001	7.69	11.48
	2012	8.508*	0.497	<.001	6.69	10.33
	2013	7.023*	0.555	<.001	4.99	9.05
	2014	8.786*	0.496	<.001	6.97	10.6
	2015	11.163*	0.528	<.001	9.23	13.1
	2016	19.234*	0.599	<.001	17.04	21.43
	2017	17.845*	0.848	<.001	14.73	20.96
1999	1990	-1.48	0.93	0.997	-4.9	1.94
	1991	-9.872*	1.448	<.001	-15.29	-4.45
	1992	19.435*	1.853	<.001	9.79	29.08
	1996	6.71	3.28	0.876	-9.28	22.7
	1997	-9.181*	1.711	<.001	-15.6	-2.77
	1998	-6.954*	0.46	<.001	-8.64	-5.27
	2000	-0.499	0.497	1	-2.32	1.32
	2001	-5.858*	0.729	<.001	-8.53	-3.18
	2002	2.862	1.803	0.997	-3.93	9.65
	2003	9.420*	0.698	<.001	6.86	11.98
	2004	14.036*	0.531	<.001	12.09	15.98
	2005	15.515*	0.437	<.001	13.92	17.11
	2006	19.277*	0.41	<.001	17.78	20.78
	2007	15.876*	0.411	<.001	14.37	17.38
	2008	13.654*	0.415	<.001	12.13	15.17
	2009	8.165*	0.423	<.001	6.62	9.71
	2010	7.754*	0.415	<.001	6.24	9.27
	2011	2.631*	0.462	<.001	0.94	4.32
	2012	1.554	0.438	0.072	-0.05	3.16
	2013	0.07	0.503	1	-1.77	1.91
	2014	1.832*	0.438	0.007	0.23	3.44
	2015	4.209*	0.473	<.001	2.48	5.94
	2016	12.281*	0.552	<.001	10.26	14.3
	2017	10.891*	0.815	<.001	7.89	13.89
2000	1990	-0.982	0.977	1	-4.57	2.61
	1991	-9.373*	1.478	<.001	-14.9	-3.85
	1992	19.933*	1.877	<.001	10.32	29.54
	1996	7.208	3.293	0.821	-8.77	23.19
	1997	-8.683*	1.737	<.001	-15.19	-2.18
	1998	-6.455*	0.549	<.001	-8.47	-4.44

	1999	0.499	0.497	1	-1.32	2.32
	2001	-5.360*	0.788	<.001	-8.25	-2.47
	2002	3.361	1.828	0.978	-3.51	10.23
	2003	9.919*	0.759	<.001	7.13	12.7
	2004	14.534*	0.609	<.001	12.3	16.76
	2005	16.013*	0.529	<.001	14.08	17.95
	2006	19.776*	0.508	<.001	17.92	21.63
	2007	16.375*	0.508	<.001	14.51	18.24
	2008	14.152*	0.512	<.001	12.28	16.03
	2009	8.663*	0.518	<.001	6.77	10.56
	2010	8.252*	0.512	<.001	6.38	10.13
	2011	3.129*	0.551	<.001	1.11	5.14
	2012	2.053*	0.531	0.024	0.11	4
	2013	0.568	0.585	1	-1.58	2.71
	2014	2.331*	0.531	0.003	0.39	4.27
	2015	4.708*	0.56	<.001	2.66	6.76
	2016	12.779*	0.628	<.001	10.48	15.08
	2017	11.390*	0.869	<.001	8.2	14.58
2001	1990	4.378*	1.113	0.02	0.29	8.46
	1991	-4.014	1.571	0.653	-9.86	1.83
	1992	25.293*	1.951	<.001	15.75	34.84
	1996	12.568	3.336	0.181	-3.41	28.54
	1997	-3.323	1.817	0.981	-10.1	3.45
	1998	-1.095	0.765	0.999	-3.9	1.71
	1999	5.858*	0.729	<.001	3.18	8.53
	2000	5.360*	0.788	<.001	2.47	8.25
	2002	8.720*	1.904	0.003	1.59	15.85
	2003	15.279*	0.928	<.001	11.88	18.68
	2004	19.894*	0.809	<.001	16.93	22.86
	2005	21.373*	0.751	<.001	18.62	24.13
	2006	25.135*	0.736	<.001	22.43	27.84
	2007	21.734*	0.736	<.001	19.03	24.44
	2008	19.512*	0.739	<.001	16.8	22.22
	2009	14.023*	0.743	<.001	11.3	16.75
	2010	13.612*	0.739	<.001	10.9	16.32
	2011	8.489*	0.766	<.001	5.68	11.3
	2012	7.412*	0.752	<.001	4.65	10.17
	2013	5.928*	0.791	<.001	3.02	8.83
	2014	7.691*	0.752	<.001	4.93	10.45
	2015	10.067*	0.773	<.001	7.23	12.9
	2016	18.139*	0.823	<.001	15.12	21.16
	2017	16.749*	1.019	<.001	13.01	20.49
2002	1990	-4.343	1.99	0.885	-11.77	3.08
	1991	-12.734*	2.278	<.001	-21.19	-4.27
	1992	16.572*	2.555	<.001	6.13	27.01

	1996	3.847	3.722	1	-12.44	20.14
	1997	-12.044*	2.454	<.001	-21.15	-2.94
	1998	-9.816*	1.818	<.001	-16.65	-2.98
	1999	-2.862	1.803	0.997	-9.65	3.93
	2000	-3.361	1.828	0.978	-10.23	3.51
	2001	-8.720*	1.904	0.003	-15.85	-1.59
	2003	6.558	1.893	0.111	-0.53	13.65
	2004	11.173*	1.837	<.001	4.27	18.08
	2005	12.652*	1.812	<.001	5.83	19.47
	2006	16.415*	1.806	<.001	9.62	23.21
	2007	13.014*	1.806	<.001	6.21	19.81
	2008	10.791*	1.807	<.001	3.99	17.59
	2009	5.302	1.809	0.375	-1.51	12.11
	2010	4.892	1.807	0.538	-1.91	11.69
	2011	-0.232	1.819	1	-7.07	6.61
	2012	-1.308	1.813	1	-8.13	5.51
	2013	-2.793	1.83	0.998	-9.67	4.08
	2014	-1.03	1.813	1	-7.85	5.79
	2015	1.347	1.822	1	-5.5	8.2
	2016	9.418*	1.844	<.001	2.49	16.34
	2017	8.029*	1.939	0.013	0.78	15.28
2003	1990	-10.901*	1.093	<.001	-14.91	-6.89
	1991	-19.292*	1.557	<.001	-25.09	-13.49
	1992	10.014*	1.94	0.038	0.46	19.57
	1996	-2.711	3.329	1	-18.69	13.26
	1997	-18.602*	1.805	<.001	-25.34	-11.87
	1998	-16.374*	0.736	<.001	-19.07	-13.67
	1999	-9.420*	0.698	<.001	-11.98	-6.86
	2000	-9.919*	0.759	<.001	-12.7	-7.13
	2001	-15.279*	0.928	<.001	-18.68	-11.88
	2002	-6.558	1.893	0.111	-13.65	0.53
	2004	4.615*	0.782	<.001	1.75	7.48
	2005	6.094*	0.721	<.001	3.45	8.74
	2006	9.857*	0.706	<.001	7.27	12.45
	2007	6.456*	0.706	<.001	3.86	9.05
	2008	4.233*	0.709	<.001	1.63	6.83
	2009	-1.256	0.713	0.99	-3.87	1.36
	2010	-1.667	0.708	0.799	-4.27	0.93
	2011	-6.790*	0.737	<.001	-9.49	-4.09
	2012	-7.866*	0.722	<.001	-10.52	-5.22
	2013	-9.351*	0.763	<.001	-12.15	-6.55
	2014	-7.588*	0.722	<.001	-10.24	-4.94
	2015	-5.211*	0.744	<.001	-7.94	-2.48
	2016	2.86	0.796	0.064	-0.06	5.78
	2017	1.471	0.997	0.999	-2.19	5.13

2004	1990	-15.516*	0.995	<.001	-19.17	-11.86
	1991	-23.907*	1.49	<.001	-29.47	-18.34
	1992	5.399	1.886	0.508	-4.2	15
	1996	-7.326	3.298	0.808	-23.31	8.65
	1997	-23.217*	1.747	<.001	-29.75	-16.68
	1998	-20.989*	0.58	<.001	-23.11	-18.87
	1999	-14.036*	0.531	<.001	-15.98	-12.09
	2000	-14.534*	0.609	<.001	-16.76	-12.3
	2001	-19.894*	0.809	<.001	-22.86	-16.93
	2002	-11.173*	1.837	<.001	-18.08	-4.27
	2003	-4.615*	0.782	<.001	-7.48	-1.75
	2005	1.479	0.561	0.587	-0.57	3.53
	2006	5.242*	0.54	<.001	3.26	7.22
	2007	1.84	0.541	0.112	-0.14	3.82
	2008	-0.382	0.544	1	-2.37	1.61
	2009	-5.871*	0.55	<.001	-7.88	-3.86
	2010	-6.282*	0.544	<.001	-8.27	-4.29
	2011	-11.405*	0.581	<.001	-13.53	-9.28
	2012	-12.481*	0.562	<.001	-14.54	-10.42
	2013	-13.966*	0.614	<.001	-16.21	-11.72
	2014	-12.203*	0.562	<.001	-14.26	-10.15
	2015	-9.827*	0.59	<.001	-11.99	-7.67
	2016	-1.755	0.654	0.552	-4.15	0.64
	2017	-3.144	0.888	0.076	-6.41	0.12
2005	1990	-16.995*	0.948	<.001	-20.48	-13.51
	1991	-25.387*	1.459	<.001	-30.85	-19.93
	1992	3.92	1.862	0.846	-5.71	13.55
	1996	-8.805	3.285	0.587	-24.79	7.18
	1997	-24.696*	1.721	<.001	-31.14	-18.25
	1998	-22.468*	0.495	<.001	-24.28	-20.66
	1999	-15.515*	0.437	<.001	-17.11	-13.92
	2000	-16.013*	0.529	<.001	-17.95	-14.08
	2001	-21.373*	0.751	<.001	-24.13	-18.62
	2002	-12.652*	1.812	<.001	-19.47	-5.83
	2003	-6.094*	0.721	<.001	-8.74	-3.45
	2004	-1.479	0.561	0.587	-3.53	0.57
	2006	3.762*	0.448	<.001	2.12	5.4
	2007	0.361	0.449	1	-1.28	2.01
	2008	-1.861*	0.453	0.01	-3.52	-0.2
	2009	-7.350*	0.46	<.001	-9.03	-5.67
	2010	-7.761*	0.453	<.001	-9.42	-6.1
	2011	-12.884*	0.497	<.001	-14.7	-11.07
	2012	-13.961*	0.474	<.001	-15.7	-12.22
	2013	-15.445*	0.535	<.001	-17.4	-13.49
	2014	-13.682*	0.474	<.001	-15.42	-11.95

	2015	-11.306*	0.507	<.001	-13.16	-9.45
	2016	-3.234*	0.581	<.001	-5.36	-1.11
	2017	-4.624*	0.835	<.001	-7.7	-1.55
2006	1990	-20.758*	0.936	<.001	-24.2	-17.32
	1991	-29.149*	1.451	<.001	-34.58	-23.72
	1992	0.157	1.856	1	-9.48	9.8
	1996	-12.568	3.281	0.175	-28.55	3.42
	1997	-28.459*	1.714	<.001	-34.88	-22.03
	1998	-26.231*	0.472	<.001	-27.96	-24.5
	1999	-19.277*	0.41	<.001	-20.78	-17.78
	2000	-19.776*	0.508	<.001	-21.63	-17.92
	2001	-25.135*	0.736	<.001	-27.84	-22.43
	2002	-16.415*	1.806	<.001	-23.21	-9.62
	2003	-9.857*	0.706	<.001	-12.45	-7.27
	2004	-5.242*	0.54	<.001	-7.22	-3.26
	2005	-3.762*	0.448	<.001	-5.4	-2.12
	2007	-3.401*	0.424	<.001	-4.95	-1.85
	2008	-5.624*	0.428	<.001	-7.19	-4.06
	2009	-11.113*	0.435	<.001	-12.7	-9.52
	2010	-11.523*	0.427	<.001	-13.09	-9.96
	2011	-16.647*	0.474	<.001	-18.38	-14.91
	2012	-17.723*	0.45	<.001	-19.37	-16.07
	2013	-19.208*	0.514	<.001	-21.09	-17.33
	2014	-17.445*	0.45	<.001	-19.09	-15.8
	2015	-15.068*	0.484	<.001	-16.84	-13.29
	2016	-6.997*	0.561	<.001	-9.05	-4.94
	2017	-8.386*	0.822	<.001	-11.41	-5.36
2007	1990	-17.356*	0.937	<.001	-20.8	-13.92
	1991	-25.748*	1.451	<.001	-31.18	-20.31
	1992	3.559	1.856	0.908	-6.08	13.2
	1996	-9.166	3.281	0.532	-25.15	6.82
	1997	-25.057*	1.715	<.001	-31.48	-18.63
	1998	-22.830*	0.473	<.001	-24.56	-21.1
	1999	-15.876*	0.411	<.001	-17.38	-14.37
	2000	-16.375*	0.508	<.001	-18.24	-14.51
	2001	-21.734*	0.736	<.001	-24.44	-19.03
	2002	-13.014*	1.806	<.001	-19.81	-6.21
	2003	-6.456*	0.706	<.001	-9.05	-3.86
	2004	-1.84	0.541	0.112	-3.82	0.14
	2005	-0.361	0.449	1	-2.01	1.28
	2006	3.401*	0.424	<.001	1.85	4.95
	2008	-2.222*	0.428	<.001	-3.79	-0.65
	2009	-7.711*	0.436	<.001	-9.31	-6.12
	2010	-8.122*	0.428	<.001	-9.69	-6.55
	2011	-13.245*	0.474	<.001	-14.98	-11.51

	2012	-14.322*	0.451	<.001	-15.97	-12.67
	2013	-15.806*	0.514	<.001	-17.69	-13.92
	2014	-14.044*	0.451	<.001	-15.69	-12.39
	2015	-11.667*	0.485	<.001	-13.44	-9.89
	2016	-3.595*	0.562	<.001	-5.65	-1.54
	2017	-4.985*	0.822	<.001	-8.01	-1.96
2008	1990	-15.134*	0.938	<.001	-18.58	-11.69
	1991	-23.525*	1.453	<.001	-28.96	-18.09
	1992	5.781	1.857	0.41	-3.86	15.42
	1996	-6.944	3.282	0.85	-22.93	9.04
	1997	-22.835*	1.716	<.001	-29.26	-16.41
	1998	-20.607*	0.476	<.001	-22.35	-18.86
	1999	-13.654*	0.415	<.001	-15.17	-12.13
	2000	-14.152*	0.512	<.001	-16.03	-12.28
	2001	-19.512*	0.739	<.001	-22.22	-16.8
	2002	-10.791*	1.807	<.001	-17.59	-3.99
	2003	-4.233*	0.709	<.001	-6.83	-1.63
	2004	0.382	0.544	1	-1.61	2.37
	2005	1.861*	0.453	0.01	0.2	3.52
	2006	5.624*	0.428	<.001	4.06	7.19
	2007	2.222*	0.428	<.001	0.65	3.79
	2009	-5.489*	0.44	<.001	-7.1	-3.88
	2010	-5.900*	0.432	<.001	-7.48	-4.32
	2011	-11.023*	0.478	<.001	-12.77	-9.27
	2012	-12.099*	0.455	<.001	-13.76	-10.43
	2013	-13.584*	0.518	<.001	-15.48	-11.69
	2014	-11.821*	0.455	<.001	-13.49	-10.16
	2015	-9.445*	0.489	<.001	-11.23	-7.66
	2016	-1.373	0.565	0.747	-3.44	0.7
	2017	-2.763	0.824	0.134	-5.8	0.27
2009	1990	-9.645*	0.942	<.001	-13.1	-6.19
	1991	-18.036*	1.455	<.001	-23.48	-12.59
	1992	11.270*	1.859	0.021	1.63	20.91
	1996	-1.455	3.283	1	-17.44	14.53
	1997	-17.346*	1.717	<.001	-23.78	-10.91
	1998	-15.118*	0.483	<.001	-16.89	-13.35
	1999	-8.165*	0.423	<.001	-9.71	-6.62
	2000	-8.663*	0.518	<.001	-10.56	-6.77
	2001	-14.023*	0.743	<.001	-16.75	-11.3
	2002	-5.302	1.809	0.375	-12.11	1.51
	2003	1.256	0.713	0.99	-1.36	3.87
	2004	5.871*	0.55	<.001	3.86	7.88
	2005	7.350*	0.46	<.001	5.67	9.03
	2006	11.113*	0.435	<.001	9.52	12.7
	2007	7.711*	0.436	<.001	6.12	9.31

	2008	5.489*	0.44	<.001	3.88	7.1
	2010	-0.411	0.439	1	-2.02	1.2
	2011	-5.534*	0.484	<.001	-7.31	-3.76
	2012	-6.610*	0.462	<.001	-8.3	-4.92
	2013	-8.095*	0.524	<.001	-10.01	-6.18
	2014	-6.332*	0.462	<.001	-8.02	-4.64
	2015	-3.956*	0.495	<.001	-5.77	-2.14
	2016	4.116*	0.57	<.001	2.03	6.21
	2017	2.727	0.828	0.157	-0.32	5.77
2010	1990	-9.234*	0.938	<.001	-12.68	-5.79
	1991	-17.626*	1.453	<.001	-23.06	-12.19
	1992	11.681*	1.857	0.017	2.04	21.32
	1996	-1.044	3.282	1	-17.03	14.94
	1997	-16.935*	1.716	<.001	-23.36	-10.51
	1998	-14.708*	0.476	<.001	-16.45	-12.96
	1999	-7.754*	0.415	<.001	-9.27	-6.24
	2000	-8.252*	0.512	<.001	-10.13	-6.38
	2001	-13.612*	0.739	<.001	-16.32	-10.9
	2002	-4.892	1.807	0.538	-11.69	1.91
	2003	1.667	0.708	0.799	-0.93	4.27
	2004	6.282*	0.544	<.001	4.29	8.27
	2005	7.761*	0.453	<.001	6.1	9.42
	2006	11.523*	0.427	<.001	9.96	13.09
	2007	8.122*	0.428	<.001	6.55	9.69
	2008	5.900*	0.432	<.001	4.32	7.48
	2009	0.411	0.439	1	-1.2	2.02
	2011	-5.123*	0.478	<.001	-6.87	-3.37
	2012	-6.200*	0.455	<.001	-7.86	-4.54
	2013	-7.684*	0.517	<.001	-9.58	-5.79
	2014	-5.921*	0.455	<.001	-7.59	-4.26
	2015	-3.545*	0.488	<.001	-5.33	-1.76
	2016	4.527*	0.565	<.001	2.46	6.6
	2017	3.137*	0.824	0.033	0.1	6.17
2011	1990	-4.111*	0.96	0.005	-7.64	-0.58
	1991	-12.502*	1.467	<.001	-17.99	-7.02
	1992	16.804*	1.868	0.002	7.18	26.43
	1996	4.079	3.288	0.999	-11.9	20.06
	1997	-11.812*	1.728	<.001	-18.28	-5.34
	1998	-9.584*	0.518	<.001	-11.48	-7.69
	1999	-2.631*	0.462	<.001	-4.32	-0.94
	2000	-3.129*	0.551	<.001	-5.14	-1.11
	2001	-8.489*	0.766	<.001	-11.3	-5.68
	2002	0.232	1.819	1	-6.61	7.07
	2003	6.790*	0.737	<.001	4.09	9.49
	2004	11.405*	0.581	<.001	9.28	13.53

	2005	12.884*	0.497	<.001	11.07	14.7
	2006	16.647*	0.474	<.001	14.91	18.38
	2007	13.245*	0.474	<.001	11.51	14.98
	2008	11.023*	0.478	<.001	9.27	12.77
	2009	5.534*	0.484	<.001	3.76	7.31
	2010	5.123*	0.478	<.001	3.37	6.87
	2012	-1.076	0.498	0.902	-2.9	0.75
	2013	-2.561*	0.556	0.001	-4.6	-0.52
	2014	-0.798	0.498	0.997	-2.62	1.03
	2015	1.579	0.529	0.322	-0.36	3.52
	2016	9.650*	0.601	<.001	7.45	11.85
	2017	8.261*	0.849	<.001	5.14	11.38
2012	1990	-3.035	0.949	0.198	-6.52	0.45
	1991	-11.426*	1.459	<.001	-16.89	-5.96
	1992	17.880*	1.862	0.001	8.25	27.51
	1996	5.155	3.285	0.983	-10.83	21.14
	1997	-10.736*	1.721	<.001	-17.18	-4.29
	1998	-8.508*	0.497	<.001	-10.33	-6.69
	1999	-1.554	0.438	0.072	-3.16	0.05
	2000	-2.053*	0.531	0.024	-4	-0.11
	2001	-7.412*	0.752	<.001	-10.17	-4.65
	2002	1.308	1.813	1	-5.51	8.13
	2003	7.866*	0.722	<.001	5.22	10.52
	2004	12.481*	0.562	<.001	10.42	14.54
	2005	13.961*	0.474	<.001	12.22	15.7
	2006	17.723*	0.45	<.001	16.07	19.37
	2007	14.322*	0.451	<.001	12.67	15.97
	2008	12.099*	0.455	<.001	10.43	13.76
	2009	6.610*	0.462	<.001	4.92	8.3
	2010	6.200*	0.455	<.001	4.54	7.86
	2011	1.076	0.498	0.902	-0.75	2.9
	2013	-1.485	0.536	0.482	-3.45	0.48
	2014	0.278	0.476	1	-1.46	2.02
	2015	2.655*	0.508	<.001	0.79	4.52
	2016	10.726*	0.582	<.001	8.59	12.86
	2017	9.337*	0.836	<.001	6.26	12.41
2013	1990	-1.55	0.98	0.998	-5.15	2.05
	1991	-9.941*	1.48	<.001	-15.47	-4.41
	1992	19.365*	1.879	<.001	9.76	28.97
	1996	6.64	3.294	0.887	-9.34	22.62
	1997	-9.251*	1.739	<.001	-15.76	-2.74
	1998	-7.023*	0.555	<.001	-9.05	-4.99
	1999	-0.07	0.503	1	-1.91	1.77
	2000	-0.568	0.585	1	-2.71	1.58
	2001	-5.928*	0.791	<.001	-8.83	-3.02

	2002	2.793	1.83	0.998	-4.08	9.67
	2003	9.351*	0.763	<.001	6.55	12.15
	2004	13.966*	0.614	<.001	11.72	16.21
	2005	15.445*	0.535	<.001	13.49	17.4
	2006	19.208*	0.514	<.001	17.33	21.09
	2007	15.806*	0.514	<.001	13.92	17.69
	2008	13.584*	0.518	<.001	11.69	15.48
	2009	8.095*	0.524	<.001	6.18	10.01
	2010	7.684*	0.517	<.001	5.79	9.58
	2011	2.561*	0.556	0.001	0.52	4.6
	2012	1.485	0.536	0.482	-0.48	3.45
	2014	1.763	0.536	0.155	-0.2	3.73
	2015	4.139*	0.565	<.001	2.07	6.21
	2016	12.211*	0.632	<.001	9.89	14.53
	2017	10.822*	0.872	<.001	7.62	14.03
2014	1990	-3.313	0.949	0.089	-6.8	0.17
	1991	-11.704*	1.459	<.001	-17.17	-6.24
	1992	17.602*	1.862	0.001	7.97	27.23
	1996	4.877	3.285	0.99	-11.11	20.86
	1997	-11.014*	1.721	<.001	-17.46	-4.57
	1998	-8.786*	0.496	<.001	-10.6	-6.97
	1999	-1.832*	0.438	0.007	-3.44	-0.23
	2000	-2.331*	0.531	0.003	-4.27	-0.39
	2001	-7.691*	0.752	<.001	-10.45	-4.93
	2002	1.03	1.813	1	-5.79	7.85
	2003	7.588*	0.722	<.001	4.94	10.24
	2004	12.203*	0.562	<.001	10.15	14.26
	2005	13.682*	0.474	<.001	11.95	15.42
	2006	17.445*	0.45	<.001	15.8	19.09
	2007	14.044*	0.451	<.001	12.39	15.69
	2008	11.821*	0.455	<.001	10.16	13.49
	2009	6.332*	0.462	<.001	4.64	8.02
	2010	5.921*	0.455	<.001	4.26	7.59
	2011	0.798	0.498	0.997	-1.03	2.62
	2012	-0.278	0.476	1	-2.02	1.46
	2013	-1.763	0.536	0.155	-3.73	0.2
	2015	2.377*	0.508	<.001	0.51	4.24
	2016	10.448*	0.582	<.001	8.32	12.58
	2017	9.059*	0.836	<.001	5.98	12.13
2015	1990	-5.689*	0.966	<.001	-9.24	-2.14
	1991	-14.081*	1.47	<.001	-19.58	-8.58
	1992	15.226*	1.871	0.003	5.61	24.84
	1996	2.501	3.29	1	-13.48	18.48
	1997	-13.390*	1.731	<.001	-19.87	-6.91
	1998	-11.163*	0.528	<.001	-13.1	-9.23

	1999	-4.209*	0.473	<.001	-5.94	-2.48
	2000	-4.708*	0.56	<.001	-6.76	-2.66
	2001	-10.067*	0.773	<.001	-12.9	-7.23
	2002	-1.347	1.822	1	-8.2	5.5
	2003	5.211*	0.744	<.001	2.48	7.94
	2004	9.827*	0.59	<.001	7.67	11.99
	2005	11.306*	0.507	<.001	9.45	13.16
	2006	15.068*	0.484	<.001	13.29	16.84
	2007	11.667*	0.485	<.001	9.89	13.44
	2008	9.445*	0.489	<.001	7.66	11.23
	2009	3.956*	0.495	<.001	2.14	5.77
	2010	3.545*	0.488	<.001	1.76	5.33
	2011	-1.579	0.529	0.322	-3.52	0.36
	2012	-2.655*	0.508	<.001	-4.52	-0.79
	2013	-4.139*	0.565	<.001	-6.21	-2.07
	2014	-2.377*	0.508	<.001	-4.24	-0.51
	2016	8.071*	0.609	<.001	5.84	10.3
	2017	6.682*	0.855	<.001	3.54	9.83
2016	1990	-13.761*	1.006	<.001	-17.45	-10.07
	1991	-22.152*	1.497	<.001	-27.74	-16.56
	1992	7.154	1.892	0.203	-2.44	16.75
	1996	-5.571	3.302	0.968	-21.55	10.41
	1997	-21.462*	1.754	<.001	-28.02	-14.9
	1998	-19.234*	0.599	<.001	-21.43	-17.04
	1999	-12.281*	0.552	<.001	-14.3	-10.26
	2000	-12.779*	0.628	<.001	-15.08	-10.48
	2001	-18.139*	0.823	<.001	-21.16	-15.12
	2002	-9.418*	1.844	<.001	-16.34	-2.49
	2003	-2.86	0.796	0.064	-5.78	0.06
	2004	1.755	0.654	0.552	-0.64	4.15
	2005	3.234*	0.581	<.001	1.11	5.36
	2006	6.997*	0.561	<.001	4.94	9.05
	2007	3.595*	0.562	<.001	1.54	5.65
	2008	1.373	0.565	0.747	-0.7	3.44
	2009	-4.116*	0.57	<.001	-6.21	-2.03
	2010	-4.527*	0.565	<.001	-6.6	-2.46
	2011	-9.650*	0.601	<.001	-11.85	-7.45
	2012	-10.726*	0.582	<.001	-12.86	-8.59
	2013	-12.211*	0.632	<.001	-14.53	-9.89
	2014	-10.448*	0.582	<.001	-12.58	-8.32
	2015	-8.071*	0.609	<.001	-10.3	-5.84
	2017	-1.389	0.901	0.998	-4.7	1.92
2017	1990	-12.372*	1.172	<.001	-16.67	-8.07
	1991	-20.763*	1.613	<.001	-26.76	-14.77
	1992	8.543	1.985	0.094	-0.99	18.08

1996	-4.182	3.356	0.999	-20.16	11.79
1997	-20.073*	1.854	<.001	-26.98	-13.17
1998	-17.845*	0.848	<.001	-20.96	-14.73
1999	-10.891*	0.815	<.001	-13.89	-7.89
2000	-11.390*	0.869	<.001	-14.58	-8.2
2001	-16.749*	1.019	<.001	-20.49	-13.01
2002	-8.029*	1.939	0.013	-15.28	-0.78
2003	-1.471	0.997	0.999	-5.13	2.19
2004	3.144	0.888	0.076	-0.12	6.41
2005	4.624*	0.835	<.001	1.55	7.7
2006	8.386*	0.822	<.001	5.36	11.41
2007	4.985*	0.822	<.001	1.96	8.01
2008	2.763	0.824	0.134	-0.27	5.8
2009	-2.727	0.828	0.157	-5.77	0.32
2010	-3.137*	0.824	0.033	-6.17	-0.1
2011	-8.261*	0.849	<.001	-11.38	-5.14
2012	-9.337*	0.836	<.001	-12.41	-6.26
2013	-10.822*	0.872	<.001	-14.03	-7.62
2014	-9.059*	0.836	<.001	-12.13	-5.98
2015	-6.682*	0.855	<.001	-9.83	-3.54
2016	1.389	0.901	0.998	-1.92	4.7

* The difference between means is significative at a level of 0.05.

Table S3. Games-Howell multiple comparisons of fork length (FL, cm) from annual scientific surveys data.

Year (I)	Year (J)	Mean difference (I-J)	Standard error	Sig.	Lower 95% confidence interval	Upper 95% confidence interval
1996	1997	-17.756*	2.052	<.001	-25.01	-10.5
	1999	-8.113*	1.772	<.001	-14.38	-1.84
	2000	0.87	1.895	1	-5.84	7.58
	2001	14.753	9.462	0.921	-67.75	97.25
	2002	6.215	6.413	0.998	-37.15	49.58
	2003	14.878*	2.955	<.001	3.99	25.77
	2004	25.887*	1.877	<.001	19.23	32.54
	2005	27.519*	1.986	<.001	20.46	34.57
	2007	25.810*	1.672	<.001	19.89	31.73
	2008	23.883*	1.653	<.001	18.03	29.73
	2010	21.111*	1.807	<.001	14.72	27.51
	2011	19.324*	1.758	<.001	13.11	25.54
	2012	22.733*	1.943	<.001	15.86	29.6
	2013	7.538*	1.749	0.003	1.35	13.73
	2016	13.886	3.712	0.078	-0.82	28.59
	2017	-1.128	6.78	1	-46.23	43.97
	2018	38.328*	3.192	<.001	26.54	50.12
	2019	27.212*	2.698	<.001	17.25	37.18
1997	1996	17.756*	2.052	<.001	10.5	25.01
	1999	9.643*	1.78	<.001	3.34	15.94
	2000	18.626*	1.902	<.001	11.89	25.37
	2001	32.509	9.463	0.392	-49.96	114.97
	2002	23.971	6.416	0.279	-19.38	67.32
	2003	32.634*	2.96	<.001	21.73	43.54
	2004	43.643*	1.885	<.001	36.96	50.32
	2005	45.275*	1.993	<.001	38.19	52.36
	2007	43.566*	1.68	<.001	37.61	49.52
	2008	41.639*	1.661	<.001	35.75	47.52
	2010	38.867*	1.814	<.001	32.44	45.29
	2011	37.080*	1.766	<.001	30.83	43.33
	2012	40.489*	1.951	<.001	33.59	47.39
	2013	25.294*	1.757	<.001	19.07	31.51
	2016	31.642*	3.716	<.001	16.93	46.36
	2017	16.628	6.782	0.634	-28.45	61.71
	2018	56.084*	3.196	<.001	44.28	67.89
	2019	44.968*	2.703	<.001	34.99	54.95
1999	1996	8.113*	1.772	<.001	1.84	14.38
	1997	-9.643*	1.78	<.001	-15.94	-3.34
	2000	8.984*	1.596	<.001	3.31	14.66
	2001	22.866	9.406	0.655	-60.91	106.65
	2002	14.328	6.332	0.706	-29.94	58.6

	2003	22.991*	2.773	<.001	12.64	33.34
	2004	34.000*	1.576	<.001	28.39	39.61
	2005	35.633*	1.704	<.001	29.55	41.72
	2007	33.923*	1.325	<.001	29.22	38.62
	2008	31.997*	1.3	<.001	27.38	36.61
	2010	29.224*	1.491	<.001	23.94	34.51
	2011	27.437*	1.432	<.001	22.37	32.51
	2012	30.847*	1.654	<.001	24.99	36.7
	2013	15.652*	1.42	<.001	10.62	20.69
	2016	21.999*	3.569	<.001	7.58	36.42
	2017	6.986	6.703	0.996	-38.91	52.88
	2018	46.441*	3.024	<.001	35.14	57.74
	2019	35.325*	2.497	<.001	25.95	44.7
2000	1996	-0.87	1.895	1	-7.58	5.84
	1997	-18.626*	1.902	<.001	-25.37	-11.89
	1999	-8.984*	1.596	<.001	-14.66	-3.31
	2001	13.882	9.43	0.94	-69.33	97.1
	2002	5.344	6.367	0.999	-38.52	49.21
	2003	14.008*	2.853	0.001	3.42	24.6
	2004	25.017*	1.712	<.001	18.92	31.12
	2005	26.649*	1.831	<.001	20.11	33.19
	2007	24.940*	1.485	<.001	19.65	30.23
	2008	23.013*	1.463	<.001	17.8	28.23
	2010	20.241*	1.635	<.001	14.43	26.06
	2011	18.453*	1.581	<.001	12.83	24.07
	2012	21.863*	1.785	<.001	15.53	28.19
	2013	6.668*	1.57	0.004	1.08	12.26
	2016	13.015	3.631	0.112	-1.53	27.56
	2017	-1.998	6.736	1	-47.54	43.54
	2018	37.458*	3.098	<.001	25.94	48.97
	2019	26.341*	2.586	<.001	16.7	35.98
2001	1996	-14.753	9.462	0.921	-97.25	67.75
	1997	-32.509	9.463	0.392	-114.97	49.96
	1999	-22.866	9.406	0.655	-106.65	60.91
	2000	-13.882	9.43	0.94	-97.1	69.33
	2002	-8.538	11.246	1	-75.83	58.75
	2003	0.125	9.699	1	-77.62	77.87
	2004	11.134	9.427	0.983	-72.16	94.43
	2005	12.766	9.449	0.962	-70.02	95.55
	2007	11.057	9.388	0.983	-73.16	95.28
	2008	9.13	9.385	0.996	-75.17	93.44
	2010	6.358	9.413	1	-77.27	89.98
	2011	4.571	9.404	1	-79.27	88.41
	2012	7.981	9.44	0.999	-75.01	90.97
	2013	-7.214	9.402	1	-91.1	76.67

	2016	-0.867	9.956	1	-74.65	72.92
	2017	-15.88	11.459	0.974	-82.85	51.09
	2018	23.575	9.774	0.656	-52.88	100.04
	2019	12.459	9.624	0.972	-66.67	91.59
2002	1996	-6.215	6.413	0.998	-49.58	37.15
	1997	-23.971	6.416	0.279	-67.32	19.38
	1999	-14.328	6.332	0.706	-58.6	29.94
	2000	-5.344	6.367	0.999	-49.21	38.52
	2001	8.538	11.246	1	-58.75	75.83
	2003	8.663	6.759	0.984	-31.94	49.27
	2004	19.672	6.362	0.43	-24.25	63.59
	2005	21.305	6.395	0.366	-22.26	64.87
	2007	19.595	6.305	0.43	-25	64.19
	2008	17.669	6.299	0.519	-26.99	62.32
	2010	14.897	6.341	0.674	-29.26	59.05
	2011	13.109	6.328	0.775	-31.2	57.42
	2012	16.519	6.382	0.587	-27.18	60.22
	2013	1.324	6.325	1	-43.02	45.67
	2016	7.671	7.122	0.997	-31.41	46.75
	2017	-7.342	9.106	1	-53.49	38.81
	2018	32.113	6.866	0.114	-7.89	72.11
	2019	20.997	6.65	0.391	-20.33	62.33
2003	1996	-14.878*	2.955	<.001	-25.77	-3.99
	1997	-32.634*	2.96	<.001	-43.54	-21.73
	1999	-22.991*	2.773	<.001	-33.34	-12.64
	2000	-14.008*	2.853	0.001	-24.6	-3.42
	2001	-0.125	9.699	1	-77.87	77.62
	2002	-8.663	6.759	0.984	-49.27	31.94
	2004	11.009*	2.842	0.033	0.45	21.57
	2005	12.641*	2.915	0.008	1.86	23.42
	2007	10.932*	2.71	0.024	0.76	21.11
	2008	9.005	2.699	0.134	-1.14	19.15
	2010	6.233	2.795	0.747	-4.19	16.65
	2011	4.446	2.765	0.978	-5.88	14.77
	2012	7.855	2.886	0.414	-2.83	18.54
	2013	-7.34	2.758	0.458	-17.65	2.97
	2016	-0.992	4.281	1	-17.28	15.29
	2017	-16.006	7.108	0.709	-58.58	26.57
	2018	23.450*	3.839	<.001	9.4	37.5
	2019	12.334	3.439	0.063	-0.3	24.97
2004	1996	-25.887*	1.877	<.001	-32.54	-19.23
	1997	-43.643*	1.885	<.001	-50.32	-36.96
	1999	-34.000*	1.576	<.001	-39.61	-28.39
	2000	-25.017*	1.712	<.001	-31.12	-18.92
	2001	-11.134	9.427	0.983	-94.43	72.16

	2002	-19.672	6.362	0.43	-63.59	24.25
	2003	-11.009*	2.842	0.033	-21.57	-0.45
	2005	1.632	1.813	1	-4.84	8.11
	2007	-0.077	1.462	1	-5.29	5.14
	2008	-2.004	1.44	0.997	-7.14	3.14
	2010	-4.776	1.614	0.247	-10.52	0.97
	2011	-6.563*	1.56	0.005	-12.11	-1.01
	2012	-3.154	1.766	0.956	-9.42	3.11
	2013	-18.349*	1.55	<.001	-23.87	-12.83
	2016	-12.001	3.622	0.182	-26.53	2.52
	2017	-27.015	6.732	0.234	-72.6	18.57
	2018	12.441*	3.087	0.022	0.95	23.93
	2019	1.325	2.573	1	-8.28	10.93
2005	1996	-27.519*	1.986	<.001	-34.57	-20.46
	1997	-45.275*	1.993	<.001	-52.36	-38.19
	1999	-35.633*	1.704	<.001	-41.72	-29.55
	2000	-26.649*	1.831	<.001	-33.19	-20.11
	2001	-12.766	9.449	0.962	-95.55	70.02
	2002	-21.305	6.395	0.366	-64.87	22.26
	2003	-12.641*	2.915	0.008	-23.42	-1.86
	2004	-1.632	1.813	1	-8.11	4.84
	2007	-1.709	1.6	1	-7.44	4.02
	2008	-3.636	1.579	0.706	-9.3	2.03
	2010	-6.408*	1.74	0.035	-12.62	-0.2
	2011	-8.196*	1.69	<.001	-14.23	-2.16
	2012	-4.786	1.881	0.526	-11.48	1.91
	2013	-19.981*	1.68	<.001	-25.99	-13.98
	2016	-13.634	3.68	0.086	-28.28	1.01
	2017	-28.647	6.763	0.2	-73.92	16.63
	2018	10.809	3.154	0.101	-0.88	22.5
	2019	-0.307	2.653	1	-10.15	9.54
2007	1996	-25.810*	1.672	<.001	-31.73	-19.89
	1997	-43.566*	1.68	<.001	-49.52	-37.61
	1999	-33.923*	1.325	<.001	-38.62	-29.22
	2000	-24.940*	1.485	<.001	-30.23	-19.65
	2001	-11.057	9.388	0.983	-95.28	73.16
	2002	-19.595	6.305	0.43	-64.19	25
	2003	-10.932*	2.71	0.024	-21.11	-0.76
	2004	0.077	1.462	1	-5.14	5.29
	2005	1.709	1.6	1	-4.02	7.44
	2008	-1.927	1.16	0.978	-6.05	2.19
	2010	-4.699	1.37	0.073	-9.57	0.17
	2011	-6.486*	1.306	<.001	-11.11	-1.86
	2012	-3.077	1.546	0.89	-8.56	2.4
	2013	-18.271*	1.293	<.001	-22.86	-13.68

	2016	-11.924	3.52	0.169	-26.25	2.41
	2017	-26.938	6.677	0.237	-73.11	19.24
	2018	12.518*	2.967	0.015	1.38	23.66
	2019	1.402	2.427	1	-7.78	10.59
2008	1996	-23.883*	1.653	<.001	-29.73	-18.03
	1997	-41.639*	1.661	<.001	-47.52	-35.75
	1999	-31.997*	1.3	<.001	-36.61	-27.38
	2000	-23.013*	1.463	<.001	-28.23	-17.8
	2001	-9.13	9.385	0.996	-93.44	75.17
	2002	-17.669	6.299	0.519	-62.32	26.99
	2003	-9.005	2.699	0.134	-19.15	1.14
	2004	2.004	1.44	0.997	-3.14	7.14
	2005	3.636	1.579	0.706	-2.03	9.3
	2007	1.927	1.16	0.978	-2.19	6.05
	2010	-2.772	1.347	0.857	-7.56	2.01
	2011	-4.560*	1.282	0.048	-9.1	-0.02
	2012	-1.15	1.526	1	-6.56	4.26
	2013	-16.345*	1.268	<.001	-20.85	-11.84
	2016	-9.998	3.511	0.382	-24.31	4.32
	2017	-25.011	6.672	0.284	-71.24	21.22
	2018	14.445*	2.956	0.002	3.34	25.55
	2019	3.329	2.414	0.995	-5.82	12.48
2010	1996	-21.111*	1.807	<.001	-27.51	-14.72
	1997	-38.867*	1.814	<.001	-45.29	-32.44
	1999	-29.224*	1.491	<.001	-34.51	-23.94
	2000	-20.241*	1.635	<.001	-26.06	-14.43
	2001	-6.358	9.413	1	-89.98	77.27
	2002	-14.897	6.341	0.674	-59.05	29.26
	2003	-6.233	2.795	0.747	-16.65	4.19
	2004	4.776	1.614	0.247	-0.97	10.52
	2005	6.408*	1.74	0.035	0.2	12.62
	2007	4.699	1.37	0.073	-0.17	9.57
	2008	2.772	1.347	0.857	-2.01	7.56
	2011	-1.787	1.475	0.999	-7.01	3.44
	2012	1.622	1.691	1	-4.37	7.61
	2013	-13.573*	1.463	<.001	-18.77	-8.38
	2016	-7.225	3.586	0.847	-21.68	7.23
	2017	-22.239	6.712	0.371	-68.03	23.56
	2018	17.217*	3.045	<.001	5.86	28.58
	2019	6.101	2.522	0.623	-3.35	15.55
2011	1996	-19.324*	1.758	<.001	-25.54	-13.11
	1997	-37.080*	1.766	<.001	-43.33	-30.83
	1999	-27.437*	1.432	<.001	-32.51	-22.37
	2000	-18.453*	1.581	<.001	-24.07	-12.83
	2001	-4.571	9.404	1	-88.41	79.27

	2002	-13.109	6.328	0.775	-57.42	31.2
	2003	-4.446	2.765	0.978	-14.77	5.88
	2004	6.563*	1.56	0.005	1.01	12.11
	2005	8.196*	1.69	<.001	2.16	14.23
	2007	6.486*	1.306	<.001	1.86	11.11
	2008	4.560*	1.282	0.048	0.02	9.1
	2010	1.787	1.475	0.999	-3.44	7.01
	2012	3.41	1.639	0.847	-2.39	9.21
	2013	-11.785*	1.403	<.001	-16.76	-6.81
	2016	-5.438	3.562	0.98	-19.84	8.97
	2017	-20.451	6.699	0.44	-66.38	25.48
	2018	19.004*	3.016	<.001	7.73	30.28
	2019	7.888	2.488	0.19	-1.46	17.24
2012	1996	-22.733*	1.943	<.001	-29.6	-15.86
	1997	-40.489*	1.951	<.001	-47.39	-33.59
	1999	-30.847*	1.654	<.001	-36.7	-24.99
	2000	-21.863*	1.785	<.001	-28.19	-15.53
	2001	-7.981	9.44	0.999	-90.97	75.01
	2002	-16.519	6.382	0.587	-60.22	27.18
	2003	-7.855	2.886	0.414	-18.54	2.83
	2004	3.154	1.766	0.956	-3.11	9.42
	2005	4.786	1.881	0.526	-1.91	11.48
	2007	3.077	1.546	0.89	-2.4	8.56
	2008	1.15	1.526	1	-4.26	6.56
	2010	-1.622	1.691	1	-7.61	4.37
	2011	-3.41	1.639	0.847	-9.21	2.39
	2013	-15.195*	1.629	<.001	-20.96	-9.42
	2016	-8.848	3.657	0.624	-23.44	5.74
	2017	-23.861	6.75	0.318	-69.26	21.53
	2018	15.595*	3.128	0.001	3.99	27.2
	2019	4.478	2.622	0.963	-5.26	14.22
2013	1996	-7.538*	1.749	0.003	-13.73	-1.35
	1997	-25.294*	1.757	<.001	-31.51	-19.07
	1999	-15.652*	1.42	<.001	-20.69	-10.62
	2000	-6.668*	1.57	0.004	-12.26	-1.08
	2001	7.214	9.402	1	-76.67	91.1
	2002	-1.324	6.325	1	-45.67	43.02
	2003	7.34	2.758	0.458	-2.97	17.65
	2004	18.349*	1.55	<.001	12.83	23.87
	2005	19.981*	1.68	<.001	13.98	25.99
	2007	18.271*	1.293	<.001	13.68	22.86
	2008	16.345*	1.268	<.001	11.84	20.85
	2010	13.573*	1.463	<.001	8.38	18.77
	2011	11.785*	1.403	<.001	6.81	16.76
	2012	15.195*	1.629	<.001	9.42	20.96

	2016	6.347	3.557	0.931	-8.05	20.74
	2017	-8.666	6.697	0.979	-54.63	37.29
	2018	30.790*	3.011	<.001	19.53	42.05
	2019	19.673*	2.481	<.001	10.34	29.01
2016	1996	-13.886	3.712	0.078	-28.59	0.82
	1997	-31.642*	3.716	<.001	-46.36	-16.93
	1999	-21.999*	3.569	<.001	-36.42	-7.58
	2000	-13.015	3.631	0.112	-27.56	1.53
	2001	0.867	9.956	1	-72.92	74.65
	2002	-7.671	7.122	0.997	-46.75	31.41
	2003	0.992	4.281	1	-15.29	17.28
	2004	12.001	3.622	0.182	-2.52	26.53
	2005	13.634	3.68	0.086	-1.01	28.28
	2007	11.924	3.52	0.169	-2.41	26.25
	2008	9.998	3.511	0.382	-4.32	24.31
	2010	7.225	3.586	0.847	-7.23	21.68
	2011	5.438	3.562	0.98	-8.97	19.84
	2012	8.848	3.657	0.624	-5.74	23.44
	2013	-6.347	3.557	0.931	-20.74	8.05
	2017	-15.013	7.454	0.809	-56.1	26.07
	2018	24.442*	4.448	<.001	7.64	41.24
	2019	13.326	4.108	0.179	-2.46	29.12
2017	1996	1.128	6.78	1	-43.97	46.23
	1997	-16.628	6.782	0.634	-61.71	28.45
	1999	-6.986	6.703	0.996	-52.88	38.91
	2000	1.998	6.736	1	-43.54	47.54
	2001	15.88	11.459	0.974	-51.09	82.85
	2002	7.342	9.106	1	-38.81	53.49
	2003	16.006	7.108	0.709	-26.57	58.58
	2004	27.015	6.732	0.234	-18.57	72.6
	2005	28.647	6.763	0.2	-16.63	73.92
	2007	26.938	6.677	0.237	-19.24	73.11
	2008	25.011	6.672	0.284	-21.22	71.24
	2010	22.239	6.712	0.371	-23.56	68.03
	2011	20.451	6.699	0.44	-25.48	66.38
	2012	23.861	6.75	0.318	-21.53	69.26
	2013	8.666	6.697	0.979	-37.29	54.63
	2016	15.013	7.454	0.809	-26.07	56.1
	2018	39.456	7.21	0.064	-2.54	81.45
	2019	28.339	7.005	0.204	-14.91	71.59
2018	1996	-38.328*	3.192	<.001	-50.12	-26.54
	1997	-56.084*	3.196	<.001	-67.89	-44.28
	1999	-46.441*	3.024	<.001	-57.74	-35.14
	2000	-37.458*	3.098	<.001	-48.97	-25.94
	2001	-23.575	9.774	0.656	-100.04	52.88

	2002	-32.113	6.866	0.114	-72.11	7.89
	2003	-23.450*	3.839	<.001	-37.5	-9.4
	2004	-12.441*	3.087	0.022	-23.93	-0.95
	2005	-10.809	3.154	0.101	-22.5	0.88
	2007	-12.518*	2.967	0.015	-23.66	-1.38
	2008	-14.445*	2.956	0.002	-25.55	-3.34
	2010	-17.217*	3.045	<.001	-28.58	-5.86
	2011	-19.004*	3.016	<.001	-30.28	-7.73
	2012	-15.595*	3.128	0.001	-27.2	-3.99
	2013	-30.790*	3.011	<.001	-42.05	-19.53
	2016	-24.442*	4.448	<.001	-41.24	-7.64
	2017	-39.456	7.21	0.064	-81.45	2.54
	2019	-11.116	3.645	0.225	-24.5	2.27
2019	1996	-27.212*	2.698	<.001	-37.18	-17.25
	1997	-44.968*	2.703	<.001	-54.95	-34.99
	1999	-35.325*	2.497	<.001	-44.7	-25.95
	2000	-26.341*	2.586	<.001	-35.98	-16.7
	2001	-12.459	9.624	0.972	-91.59	66.67
	2002	-20.997	6.65	0.391	-62.33	20.33
	2003	-12.334	3.439	0.063	-24.97	0.3
	2004	-1.325	2.573	1	-10.93	8.28
	2005	0.307	2.653	1	-9.54	10.15
	2007	-1.402	2.427	1	-10.59	7.78
	2008	-3.329	2.414	0.995	-12.48	5.82
	2010	-6.101	2.522	0.623	-15.55	3.35
	2011	-7.888	2.488	0.19	-17.24	1.46
	2012	-4.478	2.622	0.963	-14.22	5.26
	2013	-19.673*	2.481	<.001	-29.01	-10.34
	2016	-13.326	4.108	0.179	-29.12	2.46
	2017	-28.339	7.005	0.204	-71.59	14.91
	2018	11.116	3.645	0.225	-2.27	24.5

*The mean difference is significative at the level of 0.05.

Table S4. Chi-squared test for given probabilities for sex ratio (M:F) at each fork length class.

Fork length class (cm)	Males	Females	Sex ratio	Chi-squared	Df	p-value
20-24	1	0	1:0	1	1	0.3173
35-39	1	0	1:0	2	1	0.1573
40-44	0	1	0:1	1	1	0.3173
45-49	0	1	0:1	1	1	0.3173
50-54	0	6	0:1	6	1	0.01431
55-59	4	16	0.25:1	7.2	1	0.00729
60-64	1	12	0.08:1	9.3077	1	0.002282
65-69	16	19	0.84:1	0.25714	1	0.6121
70-74	23	33	0.7:1	1.7857	1	0.1814
75-79	53	55	0.96:1	0.037037	1	0.8474
80-84	101	102	0.99:1	0.0049261	1	0.944
85-89	110	111	0.99:1	0.0045249	1	0.9464
90-94	94	157	0.6:1	15.813	1	6.993e-05
95-99	50	110	0.45:1	22.5	1	2.1e-16
100-104	45	89	0.51:1	14.448	1	0.0001441
105-109	48	83	0.58:1	9.3511	1	0.002228
110-114	39	77	0.51:1	12.448	1	0.004184
115-119	31	85	0.36:1	25.138	1	5.337e-07
120-124	25	69	0.36:1	20.596	1	5.672e-06
125-129	19	77	0.25:1	35.042	1	3.227e-09
130-134	6	68	0.09:1	51.946	1	5.705e-13
135-139	0	82	0:1	82	1	2.2e-16
140-144	1	62	0.02:1	59.063	1	1.527e-14
145-149	0	60	0:1	60	1	9.486e-15
150-154	0	36	0:1	36	1	1.973e-09
155-159	0	17	0:1	17	1	3.738e-05
160-164	0	8	0:1	8	1	0.004678
165-169	0	1	0:1	1	1	0.3173
170-174	0	4	0:1	4	1	0.0455
175-179	0	2	0:1	2	1	0.1573
185-189	0	1	0:1	1	1	0.3173

Table S5 Chi-squared test for given probabilities for sex ratio (M:F) at each depth stratum.

Depth stratum (m)	Males	Females	Sex ratio	Chi-squared	Df	p-value
51-100	22	64	0.34:1	20.512	1	< 0.01
101-150	31	67	0.46:1	13.224	1	<0.01
151-200	72	199	0.36:1	59.517	1	<0.01
201-250	126	207	0.61:1	19.703	1	< 0.01
251-300	67	146	0.46:1	29.3	1	< 0.01
301-350	110	182	0.6:1	17.7	1	< 0.01
351-400	109	311	0.35:1	97.152	1	< 2.2e-16
401-450	78	157	0.5:1	26.557	1	< 0.01
451-500	29	62	0.47:1	11.967	1	< 0.01
501-550	11	28	0.39:1	7.4103	1	<0.01
551-600	13	21	0.62:1	1.8824	1	0.1701

Table S6. Chi-squared test for given probabilities for sex ratio (M:F) at each surveyed area.

Area	Males	Females	Sex ratio	Chi-squared	Df	p-value
Island	285	385	0.74:1	14.925	1	0.0001118
Seamount	384	1059	0.36:1	315.75	1	< 2.2e-16
